# Global socio-economic losses and environmental gains from the Coronavirus pandemic

**DOI:** 10.1371/journal.pone.0235654

**Published:** 2020-07-09

**Authors:** Manfred Lenzen, Mengyu Li, Arunima Malik, Francesco Pomponi, Ya-Yen Sun, Thomas Wiedmann, Futu Faturay, Jacob Fry, Blanca Gallego, Arne Geschke, Jorge Gómez-Paredes, Keiichiro Kanemoto, Steven Kenway, Keisuke Nansai, Mikhail Prokopenko, Takako Wakiyama, Yafei Wang, Moslem Yousefzadeh

**Affiliations:** 1 Integrated Sustainability Analysis, School of Physics, The University of Sydney, Sydney, NSW, Australia; 2 Discipline of Accounting, School of Business, The University of Sydney, Sydney, NSW, Australia; 3 Resource Efficient Built Environment Lab, Edinburgh Napier University, Edinburgh, United Kingdom; 4 Business School, The University of Queensland, Brisbane, QLD, Australia; 5 School of Civil and Environmental Engineering, UNSW Sydney, Sydney, NSW, Australia; 6 Fiscal Policy Agency, Ministry of Finance of The Republic of Indonesia, Jakarta, Indonesia; 7 Research Institute for Humanity and Nature, Kyoto, Japan; 8 Centre for Big Data Research in Health, UNSW Sydney, Sydney, NSW, Australia; 9 School of Earth Sciences, Energy and Environment, Yachay Tech University, Urcuquí, Ecuador; 10 Nicholas School of the Environment, Duke University, Durham, NC, United States of America; 11 Advanced Water Management Centre, The University of Queensland, Brisbane, QLD, Australia; 12 Center for Material Cycles and Waste Management Research, National Institute for Environmental Studies, Tsukuba, Japan; 13 Centre for Complex Systems, The University of Sydney, Sydney, NSW, Australia; 14 School of Statistics, Beijing Normal University, Beijing, P.R. China; Institute for Advanced Sustainability Studies, GERMANY

## Abstract

On 3 April 2020, the Director-General of the WHO stated: “*[COVID-19] is much more than a health crisis*. *We are all aware of the profound social and economic consequences of the pandemic* (WHO, 2020)”. Such consequences are the result of counter-measures such as lockdowns, and world-wide reductions in production and consumption, amplified by cascading impacts through international supply chains. Using a global multi-regional macro-economic model, we capture direct and indirect spill-over effects in terms of social and economic losses, as well as environmental effects of the pandemic. Based on information as of May 2020, we show that global consumption losses amount to 3.8$tr, triggering significant job (147 million full-time equivalent) and income (2.1$tr) losses. Global atmospheric emissions are reduced by 2.5Gt of greenhouse gases, 0.6Mt of PM_2.5_, and 5.1Mt of SO_2_ and NO_x_. While Asia, Europe and the USA have been the most directly impacted regions, and transport and tourism the immediately hit sectors, the indirect effects transmitted along international supply chains are being felt across the entire world economy. These ripple effects highlight the intrinsic link between socio-economic and environmental dimensions, and emphasise the challenge of addressing unsustainable global patterns. How humanity reacts to this crisis will define the post-pandemic world.

## Introduction

On 9 January 2020, the World Health Organisation (WHO) first reported the outbreak of a coronavirus disease (COVID-19), caused by the severe acute respiratory syndrome coronavirus 2 (SARS-CoV-2) [1], from the Chinese city of Wuhan. By the end of January, there were more than 10,000 existing cases and more than 2,000 new confirmed cases daily, mostly in China’s Hubei province, and more than 250 people had died. Initially, the spread of the virus beyond China’s borders was slow, affecting a handful of nearby regions such as Japan, Hong Kong and Singapore. However, by the end of February, when the number of daily new cases from China decreased, infections accelerated again, this time across all continents, with major outbreaks initially in South Korea and Iran, and then across Europe and the Americas (SI 1 in S1 File) [2]. On 11 March, with more than 118,000 confirmed cases in 114 countries, and 4,291 deaths, the WHO named COVID-19 a pandemic [3].

Governments reacted to the outbreak by restricting people’s movements. By 3 April, with over 1 million confirmed cases worldwide [4], many countries implemented lockdown measures, with close to 3 billion people asked to stay at home [5], more than 1 billion people alone in India. These restrictions meant that people were unable to commute to their workplaces, and as a result, offices and factories closed. Internationally, broad entry bans were applied, and flight routes suspended.

Given the important role of large coronavirus-affected economies such as China, Europe and the USA, in global manufacturing and trade, the slowdown in these countries’ production inevitably leads to significant supply-chain interruptions, affecting especially businesses that are heavily dependent on trade, such as specialised manufacturing [6] and health care supplies [7]. Businesses may rely on inventories to bridge temporary supply shortfalls, generally for two to five weeks [6], however after stocks are depleted, ensuing declines in production will cascade throughout international supply-chain networks, affecting both downstream customers and upstream suppliers. Options for switching to alternative inputs are limited wherever these inputs are specialised and essential, such as parts for vehicles. Options for switching to alternative supply locations are also limited when production is concentrated, or when the output of many regions is reduced. This has been the case during the COVID-19 global outbreak. It is therefore crucial to put in place preparedness measures that are aimed at minimising disruptions for populations and economic losses for business [8].

Apart from profound social and economic implications [4], there was however a silver lining: the grounding of planes and shutdown of factories due to the implementation of travel bans and lockdowns had a beneficial effect on air pollution. By March, the decline in coal use by power plants, oil refining, steel manufacturing and air travel was estimated to have caused a 250 Mt decrease in CO_2_ emissions [9]. NASA and the European Space Agency reported a dramatic fall in N_2_O pollution across North-Eastern China [10] and the lowest average level of N_2_O ever recorded in India was a result of the nationwide curfew at the end of March [11]. The quantification of these wide-ranging impacts at a global scale requires an ability to capture supply-chain-driven spill-over impacts across regions and sectors for multiple indicators, otherwise the assessment is incomplete [6, 12, 13]. This ability is provided by input-output analysis [14, 15], more specifically global multi-region input-output (MRIO) analysis.

Prior work has primarily focussed on modelling the mechanisms for spread of diseases [16]. Global MRIO-based supply-chain analyses of disasters exist [17], however not of a virus pandemic. Preliminary input-output analyses have been carried out for estimating the possible impacts of the COVID-19 outbreak within China [18] and Japan [19], both using national input-output (IO) tables. A study on the economic and productivity risks resulting from the 2009 H1N1 epidemic [20] covers only a small region in the USA, and recommends examining employment and income as loss categories, which is realised in this work. Initial assessments based on MRIO tables and computable general equilibrium (CGE) analysis point to scenarios of wide-ranging decline in global gross domestic product and consumption [21, 22]. There are urgent calls to develop assessment frameworks for capturing interactions between regions and sectors to enable timely quantification of actual economic impacts, such as for the COVID-19 pandemic [23], to help guide policy responses. The positive environmental effects of the pandemic are yet to be quantified.

The aim of this study is to provide a comprehensive global estimate of how the 2020 COVID-19 pandemic in the most affected countries reduced economic activity and environmental pressures in all other countries because of globalised trade links. Our study covers reduced consumption, employment, income, emissions of greenhouse gases (GHG), PM_2.5_ and other air pollutants (SO_2_ and NO_x_). Reductions in individual national economies have amplified one another, leading to even larger economic losses and environmental gains at a global scale. Our assessment differs from previous ones in that we are not offering scenarios, but an assessment of the overall global effects based on assessments of impacts as of May 2020 (SI 4 in S1 File).

## Materials and methods

### Supply-chain analysis

The transmission of monetary or physical impacts between economic sectors and regions is called spill-over. International spill-over effects have traditionally been investigated using multi-region input-output (MRIO) analysis [24, 25]. This technique was conceived by Nobel prize Laureate Wassily Leontief before WWII [26]. Since then, it has been used extensively for tracing economic and environmental impact across complex supply-chain networks [27, 28], with high-level applications to carbon emissions, biodiversity loss, air pollution, and public health [29, 30]. Input-output databases are regularly collected by more than 100 national statistical agencies worldwide, all governed by United Nations standards [31]. The centrepiece of global MRIO data is an *N*×*N* intermediate demand matrix (**T**), mapping the connections between all industry sectors in the global economy. Contemporary MRIO databases distinguish in excess of *N*≥10,000 industries and products [32]. Seeing that industries can either supply other industries or final consumers, summing intermediate and final demand (**y**; for example households) yields total output **x** = **T1**+**y**, where the vector **1** = {1,1,…,1} is a summation operator. Defining a technical coefficient matrix $A≔T{\hat{x}}^{-1}$, with the hat symbol denoting vector diagonalization, lets us derive Leontief’s fundamental accounting identity $x=T{\hat{x}}^{-1}+y\Leftrightarrow x={\left(I-A\right)}^{-1}y$, where **I** is an identity matrix [28]. The term (**I**−**A**)^−1^ is Leontief’s famous inverse, providing information on the complex links between geographically distant producers and consumers.

### Disaster analysis

One particular strand of MRIO analysis concerned with the impacts of shocks to the economy is disaster analysis [33]. Such type of analysis considers the direct and indirect supply-chain effects of disasters resulting in loss of production and reduced business activity [34]. Several variations of the input-output framework have been developed over the years for quantifying disruptions ranging from natural events [35], extreme weather events [36] to terrorism [37] and blackouts [38]. The variations have focussed on enhancing the capability of standard input-output analysis in capturing temporal frames, such as in the regional econometric input-output model [39], and for capturing recovery times in a post-disaster world as in the inoperability input-output model (IIM) [40–42], and its uses for quantifying negative economic impacts [43]. A relatively new technique of hypothetical extraction (HEM) relies on assessing hypothetical scenarios where industries cease to operate [44], as applied for the case of shutdown of IT services in the UK [45]. Since in this work we are concerned with the quantification of *actual* impacts of a global pandemic on people’s livelihoods, we build on a disaster analysis method that focuses on post-disaster consumption possibilities [46]. This method uses a so-called event matrix Γ, where diagonal elements Γ_*ii*_ describe the relative loss of industries *i* = 1,…,*N* as a direct result of a disaster. Here, we minimise the departure ${\left(\tilde{x}-x\right)}^{2}$ of post-disaster output $\tilde{x}$ from pre-disaster output **x**, subject to two conditions [47]: First, we ask that $\tilde{x}\leq (I-\Gamma )x$. Second, we require that post-disaster final demand $\tilde{y}=(I-A)\tilde{x}\geq min(0,y_{St})$. Here, **y**_St_≤0 holds information on stocks, on which industries may draw for continuing sales despite their production downturn. The condition therefore says that final demand may not be negative for industries that do not hold any stocks. For those who do hold stocks, we ask that losses may not drive final demand so far down as to exceed the value of these stocks. The solution $\tilde{y}$ of this optimisation problem are the post-disaster consumption possibilities. We then truncate $\tilde{y}$ at **y**, using $min(\tilde{y},y)$, and thus count only consumption losses and not the relatively insignificant increases in final consumption possibilities that come about through the decrease of intermediate demand [46]. This is because we assume that increased availability of commodities will not necessarily translate into increased demand, because that demand has not existed in the pre-disaster economy. In contrast to Γ, post-disaster consumption possibilities include all spill-over effects, meaning that people in regions not directly hit by the disaster may see their consumption curtailed because of supply-chain relationships. The optimisation was carried out using the quadprog function in Matlab, which calls an interior-point algorithm.

### Economic and environmental impacts

Reduced post-disaster consumption translates into reduced employment and family incomes, but also into reduced emissions of greenhouse gases and air pollutants. Whilst some of the direct environmental consequences of the COVID-19 outbreak could be measured [9], quantifying the combined effects of all regional and sectoral spill-over cascades requires an MRIO analysis. Leontief already proposed an extension of his approach to include physical quantities [48]. This is accomplished by coupling the fundamental accounting identity with a so-called satellite account (**Q**), holding data on physical quantities for every of the *N* industries in the intermediate demand matrix **T**. Following Leontief’s calculus, the economic and environmental impacts *F* of the disaster can be computed from consumption losses–the difference of pre-and post-disaster consumption possibilities $\tilde{y}-y$ –as $F=Q{\hat{x}}^{-1}{\left(I-A\right)}^{-1}(\tilde{y}-y)$ [49]. We carry out an uncertainty analysis [49] to accompany our results with reliability estimates (SI 5 in S1 File).

### Data sources

Meaningful application of MRIO analysis to the spill-over effects of a global pandemic requires providing rapid information on regions and sectors that will likely be affected. However, many official input-output and national accounts are published with a multi-year delay. We therefore apply a recent innovation to compiling MRIO accounts: a virtual laboratory [50]. By employing collaborative cloud-computing environments, virtual MRIO laboratories have led to significant gains in research efficiencies and timely data provision. MRIO labs are currently operating in six countries [51]. Here we use the Global MRIO Lab [52] for compiling a tailored global MRIO data set, distinguishing 38 regions with 26 sectors each (SI 2 in S1 File). We use the most recent global data sources, for the monetary variables **T** and **y** from the United Nations, and for the satellite **Q** from the EU’s Joint Research Centre for emissions of greenhouse gases, PM_2.5_ and air pollutants, and from the International Labour Organisation for employment (SI 3 in S1 File). To populate the event matrix **Γ** for the COVID-19 pandemic, we carry out an extensive data collection exercise, including recent online accounts of industry losses across the world (SI 4 in S1 File). This strategy, combined with the utilisation of the Global MRIO Lab, allows us to rapidly respond to the outbreak with information on the likely magnitude and spread of the economic and environmental repercussions.

## Results

Our approach uses constrained non-linear optimisation (SI 5 in S1 File) to determine the maximum level of global consumption that is possible under given (exogenous) reductions of economic output by taking into account the effects of industry shutdowns, lockdowns and travel restrictions. The difference between this maximum level and the pre-COVID-19 world economy are consumption losses. In turn, these consumption losses set in motion supply-chain effects that ripple across the world economy, and lead to global losses of income, employment, and reductions of emissions.

On the basis of reported direct losses experienced by global businesses due to the COVID-19 pandemic, we estimate the total consumption loss, including all regional and sectoral spill-overs, to be about 3.8$tr, or 4.2% of global GDP, and comparable to the GDP of Germany. This figure carries an uncertainty (due to stochastic errors in underlying data based on media reports, published academic articles, official publications and expert opinion) of *σ* ≈ 10%, however systematic uncertainties mean that it is likely an underestimate (SI 5 in S1 File). Similarly, jobs of workers globally reduce by 147 million FTE (full-time equivalent), or 4.2% of the global workforce, with associated losses of wages and salaries of 2.1$tr, or 6.0% of global income. Global emissions of GHGs, PM_2.5_, and air pollutants also reduce, by 2.5 Gt, 0.6 Mt and 5.1 Mt, or 4.6%, 3.8% and 2.9% of the global annual totals, respectively. Reductions in GHG emissions are larger than any drop in anthropogenic emissions in human history (SI 6.5, SI11 Fig in S1 File), including when fossil-fuel CO_2_ emissions dropped by 0.46 Gt CO_2_ in 2009 due to the global financial crisis (GFC) [53], and when CO_2_ emissions from land use change dropped by 2.02 Gt CO_2_ in 1998 [53].

Spill-over effects are significant: our production layer decompositions (SI 6 in S1 File) show that direct impacts are magnified about 1.5-fold, as a consequence of the immediate impacts rippling through the global supply-chain network and causing widespread indirect impacts. In the following, we will unravel the overall results given above, and provide insights for individual regions and industry sectors.

**Fig 1:**
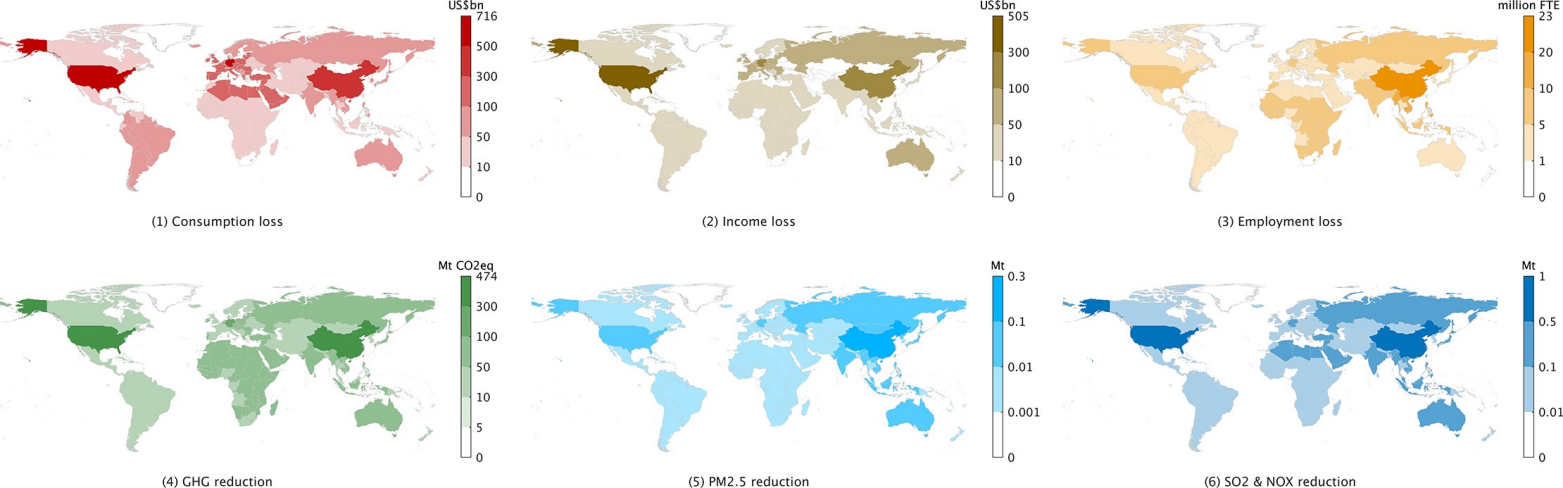
Global impacts from the COVID-19 pandemic broken down by world region. Accompanying data tables are in SI 6.1 in S1 File.

**Fig 2:**
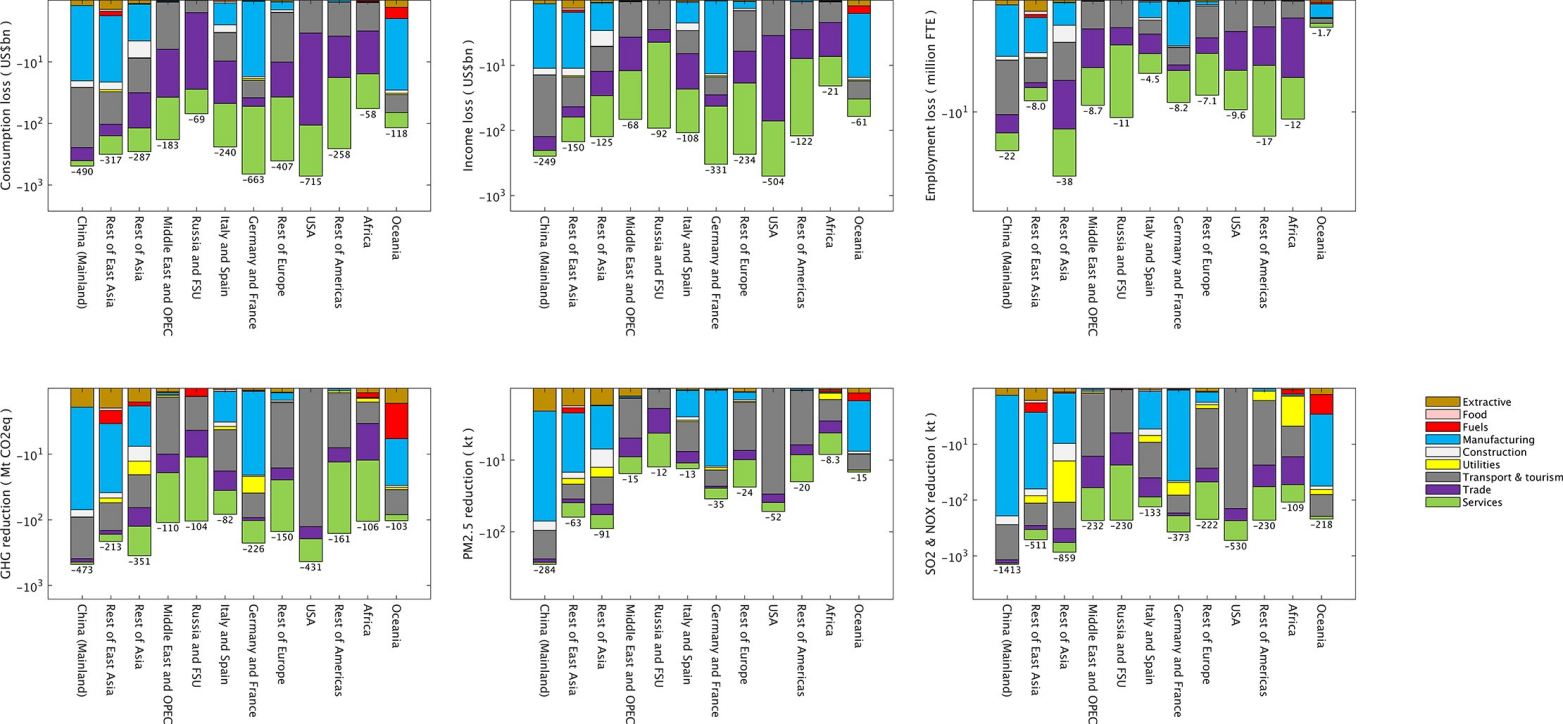
Sectoral breakdown of global impacts from the COVID-19 pandemic, in indicator-specific units (US$bn for consumption and income, million FTE for employment, Mt for greenhouse gas emissions, and kt for other emissions). The bands represent direct and indirect impacts by purchased commodity. For example, the Yellow band refers to final demand purchases of electricity, gas and water; however, utilities’ losses and reductions in income, jobs and emissions are also included in the supply chain of other commodities, such as Manufacturing (Blue). Accompanying data tables are in SI 6.2 in S1 File.

The global figures listed above can be broken down into estimates for the 38 regions distinguished in our study (SI 2 in S1 File). Clearly, the more significant consumption and income losses are borne out in large economies with either high numbers of coronavirus cases and/or stringent countermeasures, i.e. China, USA, Italy, Spain, Germany, UK and France (Fig 1). OPEC nations lose income because of reduced oil extraction and refining activity as a result of the reductions in transport, especially aviation. Low-wage countries such as China and India stand out in terms of employment losses. Reductions in GHG emissions occur all over the globe, but mainly across China and Northern America, and less across Europe’s less emissions-intensive economies. Reductions of PM_2.5_ emissions are expectedly large in China and India, whilst energy- and transport-related SO_2_ and NO_x_ emissions also fall across the rest of Asia and the Americas (Fig 2). The results presented in this study are based on current information gathered from various sources (SI 4 in S1 File), yet are broadly comparable with estimates produced by international organisations (SI 7 in S1 File).

An assessment of total impacts of COVID-19 at a sector-level reveals that transport and tourism are the economically worst-hit sectors (Fig 2). This is unsurprising, with falling air travel demands as people made cancellations, coupled with a suite of travel restrictions imposed by countries worldwide to slow the spread of the virus, and airlines going bankrupt [54]. The International Air Transport Association (IATA) estimated that global revenues could fall more than 44% below 2019 figures [55] (SI 4.4 in S1 File). Significantly affected by lockdowns are retail and wholesale, as well as service sectors, including business services in the supply chains of tourism and transport, and entertainment and personal services. Manufacturing operations are reduced in China, Europe, and across the OPEC, mining in Australia [56] (ores and gas) and the OPEC (oil), because of supply-chain knock-on effects.

Manufacturing and Transport & tourism dominate reductions in GHG and SO_2_ emissions, because of their intensive use of fuels (Fig 2). A large part of reductions in PM_2.5_ emissions globally is driven by reduced power, gas and water utilities output in Asia and the Americas. It has been previously shown that developed nations outsource PM_2.5_-related impacts to Asia [57]. The fact that utilities are not represented more uniformly across regions is either due to reductions not having occurred, or missing information. This once more underlies that our results are likely underestimates (SI 5 in S1 File).

**Fig 3:**
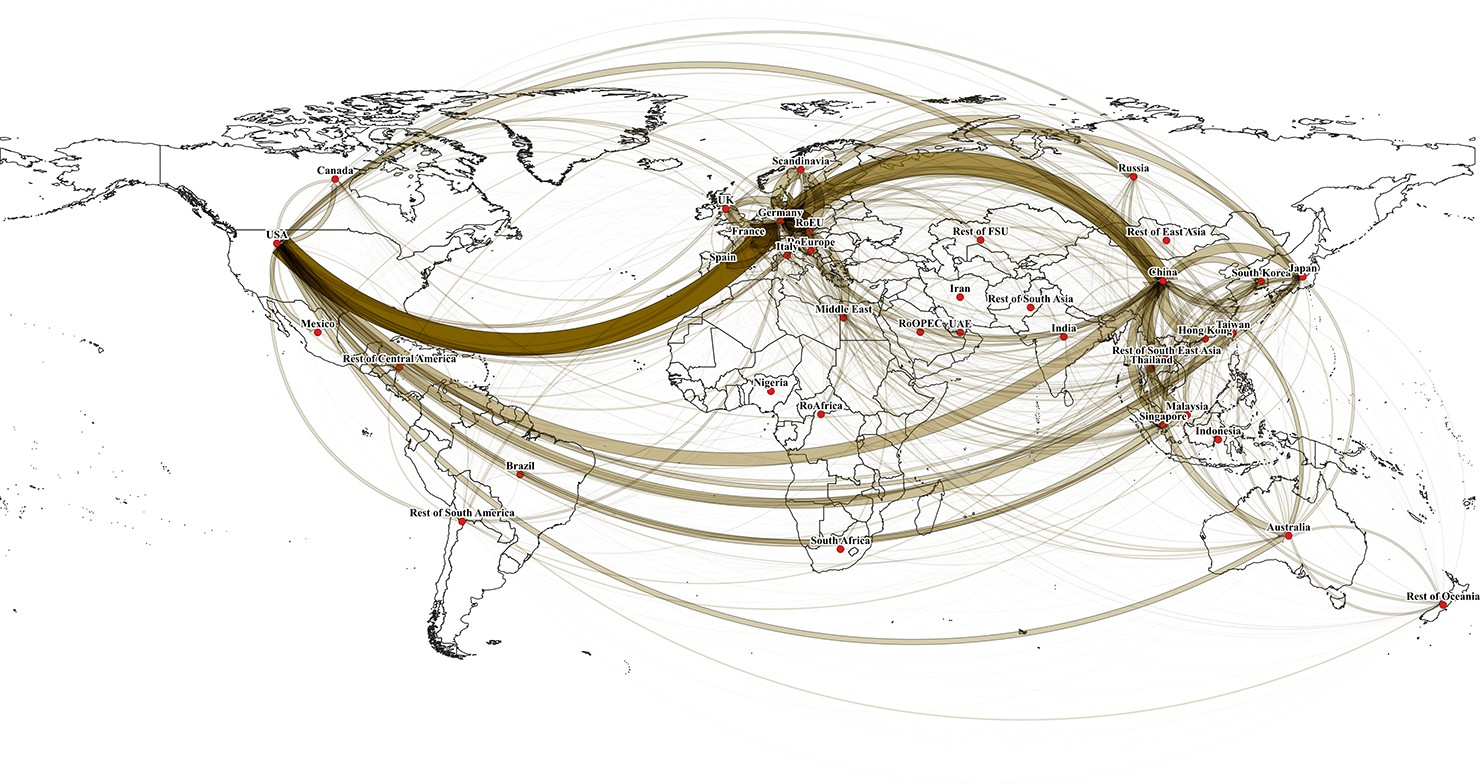
Wage and salary income losses as a consequence of trade volume reductions in international supply chains due to the global COVID-19 effects. Lines connect ultimate origins and destinations of supply chains, both direct and multi-node. Line thickness represents trade volume lost.

Out of the total income losses of $2.1tr, $536bn or about 21% is lost because of a reduction in international trade, demonstrating the importance of international spill-overs that cause the effects of the COVID-19 pandemic to be felt in all countries across the globe (Fig 3). Given the reliance of many national economies on China, we observe significant losses in supply chains that originate in Mainland China. The pandemic has exposed vulnerable businesses whose supply chains are heavily concentrated in countries that are most directly impacted by the crisis [58]. Countries that have significant trade relationships with the most coronavirus-affected countries also experience emission reductions (SI 6 in S1 File).

## Discussion

The core principles underlying the mitigation and suppression strategies adopted to control the pandemic–distancing of individuals and quarantining of communities–work directly against the strengths of the global economy, built around connectivity and inter-dependence. Ironically, the loss of connectivity imposed to prevent the spread of the COVID-19 has triggered an economic “contagion”, with the crisis precipitated by the COVID-19 pandemic cascading across socio-economic sectors, and causing major disruptions to trade, tourism, energy and finance sectors.

The global economy is heading towards a recession “way worse than the GFC” [59]; global consumption loss thus far, including both direct and indirect spill-over effects is significant. The strongest effects are felt in China, other major Asian economies, Europe, and USA, and largely in the transport and tourism sector, yet with world-wide ripple effects. These losses are likely to increase and spread to the rest of the global economy, as lockdown measures continue. Yet lifting restrictions too soon could result in more severe and prolonged economic impacts [3]. Thus, governments are faced with the challenge of attempting to keep the global economy afloat by spending the International Monetary Funds’ war chest [59] and other emergency funds, while trying to find new ways of working (e.g. extending teleworking) [60]. Global economic measures taken to address the economic impacts of COVID-19 have been rapidly developed. To-date, most measures have aimed at broad-brush support for “business”, e.g. the US, UK and Australian governments committing significant support for businesses and households. Other measures include (a) additional bank lending (b) health system improvements (largely intensive care facilities), support to State and Local government account for a large percentage of packages. Measures which directly address impacts to sectors impacted in Fig 2 (e.g. Transport & Tourism, Trade and Services) are less explicit. It appears that governments’ primary approach is to put a safety net under the economy, rather than to promote those forms of business either most affected, or most able to grow and develop in the anticipated post-COVID-19 economic landscape.

Socially, the pandemic has resulted in significant labour market shocks [60], which are poised to grow as the pandemic persists. Moreover, subsequent economic shocks are likely to impact even further the quantity and quality of jobs, as well as affect vulnerable groups [60], such as migrant and unskilled workers who may not adapt to virtual-work arrangements.

Environmentally, the pandemic has brought a reduction in greenhouse gas and air pollution, mainly from a fall in fossil fuel consumption as airplanes are grounded, transportation reduced, trade hindered, and factories closed down. These bring important environmental gains as well as social benefits. Reductions in PM_2.5_ alone are likely to save thousands of lives. The current drop in GHG emissions is larger than anything the world has seen since humans started to use fossil fuels (our estimates until the end of May 2020 are at the higher end of estimates of global CO_2_ emission reductions projected until the end of 2020 by one other study [61]). None of the attempts by any government or any international agreement in the 32-year history of intergovernmental climate policy has had such a dramatic mitigation effect. Incidentally, the drop of approximately 4.5% in global GHG emissions caused by reactions to COVID-19 still falls short of what would be needed every year until 2050 to limit global warming to 1.5ºC (SI13 Fig in S1 File).

Clearly, the social and environmental consequences of fighting the pandemic will be much broader than negative impacts on jobs and income, and positive impacts on atmospheric pollution (e.g. fighting the pandemic may bring about mass surveillance [62, 63], or negative environmental impacts from continuous cleaning and disinfecting activities [64]). However, the contrast between the socio-economic and the environmental variables that we have assessed in this study reveals the dilemma of the global socio-economic system.

The short-term economic, social, and environmental impacts of the COVID-19 pandemic are profound; and pose several challenges and dilemmas. The current crisis is likely to deepen systemic socioeconomic vulnerabilities, widen income and wealth gaps, overburden, if not decimate, healthcare systems in lower income countries [65], and proliferate the spread of emerging zoonotic diseases [66]. When such systemic vulnerabilities are coupled with significant climatic variations, their combined effects may lead to tipping points in socio-economic systems, e.g. via food systems failure and large-scale urban abandonment [67].

To this point, it is worth noting that the COVID-19 crisis takes place in the shadow of other more “silent”, yet much longer-lasting, ongoing global crises, such as climate change. The GFC led to only a small dent in the continued upward trajectory of global GHG emissions [68]. While the COVID-19 crisis will leave a larger impression, it will still not be enough to avoid dangerous climate change, and may quickly be erased as we attempt to go back to business-as-usual and give way to “retaliatory pollution” [69]. This could potentially be averted by implementing stimulus plans to boost clean energy technologies and facilitate ‘just transitions’, to encourage investments in teleworking and teleconferences for reducing carbon-intensive travel, and to devise policies for addressing rebound effects. Yet, it remains to be seen if such an approach would fare differently than the G20 countries’ green economic stimulus after the GFC [70], and if countries eager to get back to economic growth will avoid basing it on low-priced oil. For business-as-usual economic growth is still strongly coupled with the use of fossil fuels and will therefore undermine any environmental gains. In contrast, the current environmental improvements mean social and economic hardship since the unfolding global recession is unplanned and unmanaged. Scientists have long argued that any deliberate economic ‘de-growth’ intended to prevent global ecological crises needs to be well managed and based on targeted investment and tax policies [71, 72].

On one hand the pandemic has shown the risks and fragility of our highly interconnected and interdependent economies and societies, which highlights the need for global cooperation and solidarity, as no country will be “immune” to situations developing elsewhere. On the other hand, it has shown that we can face crises with concerted and decisive interventions and changes in behaviours and lifestyles, which can lead to significant environmental benefits while protecting people's livelihoods at the same time [71]. Both are key factors to address the wider environmental crises.

Future work could focus on examining the impact of lasting changes in behaviour and lifestyles induced by the pandemic on wider global issues, such as greenhouse gas and air pollutant emissions, resource use or biodiversity decline. Unequivocally, the COVID-19 pandemic has had varying levels of effects on economy and environment of low-, middle- and high-income countries, and those of islands. The future of the global ecosystem hinges on regional policy decisions that will define the post-pandemic world. Government policies during the pandemic have been reported to have caused a 17% drop in daily global emissions in April 2020, compared to the 2019 average, and up to 20% in some countries [61]. Much of these policies are temporary measures for slowing down the spread of the virus, which have had a positive impact on environment and air quality. Future research focussed on analysing the post-pandemic approach of countries to transition to greener economies will shed light on the likely challenges faced in ensuring such emission reductions are maintained or even increased with the aim of meeting the Paris goals [73] and are economically viable and socially just.

The future of the global ecosystem hinges on regional policy decisions that will define the post-pandemic world. A sustainable post-pandemic world will be defined by the collective action of all societal groups to seize this opportunity to ensure a transition to green economies instead of a ‘business-as-usual’ pathway.

As indicated by the UN Secretary General [74], humanity faces a choice: attempting to return to a business-as-usual path with more unnecessary crises, or developing a different economy that is compatible with more sustainable and resilient human societies. Clearly, the decision we take now and after the crisis will define our post-pandemic world.

## Supporting information

S1 File (DOCX)

S1 Data (XLSX)
