# Correction: Global socio-economic losses and environmental gains from the Coronavirus pandemic

**DOI:** 10.1371/journal.pone.0331926

**Published:** 2025-09-08

**Authors:** Manfred Lenzen, Mengyu Li, Arunima Malik, Francesco Pomponi, Ya-Yen Sun, Thomas Wiedmann, Futu Faturay, Jacob Fry, Blanca Gallego, Arne Geschke, Jorge Gómez-Paredes, Keiichiro Kanemoto, Steven Kenway, Keisuke Nansai, Mikhail Prokopenko, Takako Wakiyama, Yafei Wang, Moslem Yousefzadeh

S1 File was uploaded incorrectly with highlighted changes. Please view the correct S1 file below.

## Supporting information

S1 File (DOCX)

## References

[1] Lenzen M, Li M, Malik A, Pomponi F, Sun Y-Y, Wiedmann T, et al. Global socio-economic losses and environmental gains from the Coronavirus pandemic. PLoS ONE. 2020;15(7):e0235654. doi: 10.1371/journal.pone.0235654 PMC7347123 32645023

