# Supplementary material for: Global socio-economic losses and environmental gains from the Coronavirus pandemic
Source: PLoS One. 2020 Jul 9;15(7):e0235654. doi: 10.1371/journal.pone.0235654 (PMC7347123; doi:10.1371/journal.pone.0235654)
Supplement: S1 File — (DOCX) [file pone.0235654.s001.docx]

**Global socio-economic losses and environmental gains from the Coronavirus pandemic**

**SUPPLEMENTARY INFORMATION**

*Manfred Lenzen^1^, Mengyu Li^1^, Arunima Malik^1,2^*, Francesco Pomponi^3^, Ya-Yen Sun^4^, Thomas Wiedmann^5^,*

*Futu Faturay^6^, Jacob Fry^1,7^, Blanca Gallego^8^, Arne Geschke^1^, Jorge Gómez-Paredes^9,10^, Keiichiro Kanemoto^7^, Steven Kenway^11^, Keisuke Nansai^1,12^, Mikhail Prokopenko^13^, Takako Wakiyama^1^, Yafei Wang^14^, Moslem Yousefzadeh^1^*

*^1^ISA, School of Physics, The University of Sydney, NSW, Australia*

*^2^ Discipline of Accounting, School of Business, The University of Sydney, NSW*

*^3^ Resource Efficient Built Environment Lab, Edinburgh Napier University, Edinburgh UK*

*^4^ Business School, the University of Queensland, QLD, Australia*

*^5^ School of Civil and Environmental Engineering, UNSW Sydney, NSW, Australia*

*^6^ Fiscal Policy Agency, Ministry of Finance of the Republic of Indonesia, Jakarta, Indonesia*

*^7^ Research Institute for Humanity and Nature, Kyoto, Japan*

*^8^ Centre for Big Data Research in Health, UNSW Sydney, NSW, Australia*

*^9^ School of Earth Sciences, Energy and Environment, Yachay Tech University, Urcuquí, Ecuador*

*^10^ Nicholas School of the Environment, Duke University, Durham NC, USA*

*^11^ Advanced Water Management Centre, The University of Queensland, QLD, Australia*

*^12^ Center for Material Cycles and Waste Management Research, National Institute for Environmental Studies, Tsukuba, Japan*

*^13^ Centre for Complex Systems, The University of Sydney, NSW, Australia*

*^14^ School of Statistics, Beijing Normal University, 19 Xinjiekouwai Street, Beijing, P.R.China*

*^*^Corresponding author:* [*arunima.malik@sydney.edu.au*](mailto:arunima.malik@sydney.edu.au)*; +61 2 93515451*

**Table of Contents**

[**SI 1 – Data on the pandemic** 3](#_Toc41915324)

[**SI 2 – MRIO compilation, and regional and sectoral classifications** 5](#_Toc41915325)

[SI 2.1 MRIO database compilation in Virtual Labs 5](#_Toc41915326)

[SI 2.2 Regional detail used in the MRIO table of this study 6](#_Toc41915327)

[SI 2.3 Sectoral detail used in the MRIO table of this study 8](#_Toc41915328)

[**SI 3 – MRIO and satellite accounts: data sources and non-linear optimization problem** 9](#_Toc41915329)

[SI 3.1 Regional detail used in the MRIO table of this study 9](#_Toc41915330)

[SI 3.2 Uniqueness of the solution of the non-linear optimisation problem 9](#_Toc41915331)

[**SI 4 – Data for populating the event (Γ) matrix** 11](#_Toc41915332)

[SI 4.1 System boundary in space 11](#_Toc41915333)

[SI 4.2 System boundary in time 13](#_Toc41915334)

[SI 4.3 Data collection procedure and data quality indicators (DQIs) 13](#_Toc41915335)

[SI 4.4 Data streams for the **Γ** matrix 14](#_Toc41915336)

[**SI 5 – Limitations, uncertainty and sensitivity analysis** 18](#_Toc41915337)

[SI 5.1 Method uncertainty 18](#_Toc41915338)

[SI 5.2 Data uncertainty 19](#_Toc41915339)

[SI 5.3 Systematic uncertainty 20](#_Toc41915340)

[SI 5.4 Sensitivity analyses 21](#_Toc41915341)

[SI 5.5 Other limitations 23](#_Toc41915342)

[**SI 6 – Additional results** 25](#_Toc41915343)

[SI 6.1 Data table for Fig.1 25](#_Toc41915344)

[SI 6.2 Data table for Fig.2 26](#_Toc41915345)

[SI 6.3 Production layer decompositions 29](#_Toc41915346)

[SI 6.4 Average wages of lost employment 30](#_Toc41915347)

[SI 6.5 GHG emissions time series 31](#_Toc41915348)

[SI 6.6 Supply chain losses as a result of COVID-19 33](#_Toc41915349)

[**SI 7 – Comparisons with other studies** 34](#_Toc41915350)

[SI 7.1 Consumption and GDP 34](#_Toc41915351)

[SI 7.2 Exports 34](#_Toc41915352)

[SI 7.3 Employment 35](#_Toc41915353)

[**References** 36](#_Toc41915354)

# **SI 1 – Data on the pandemic**


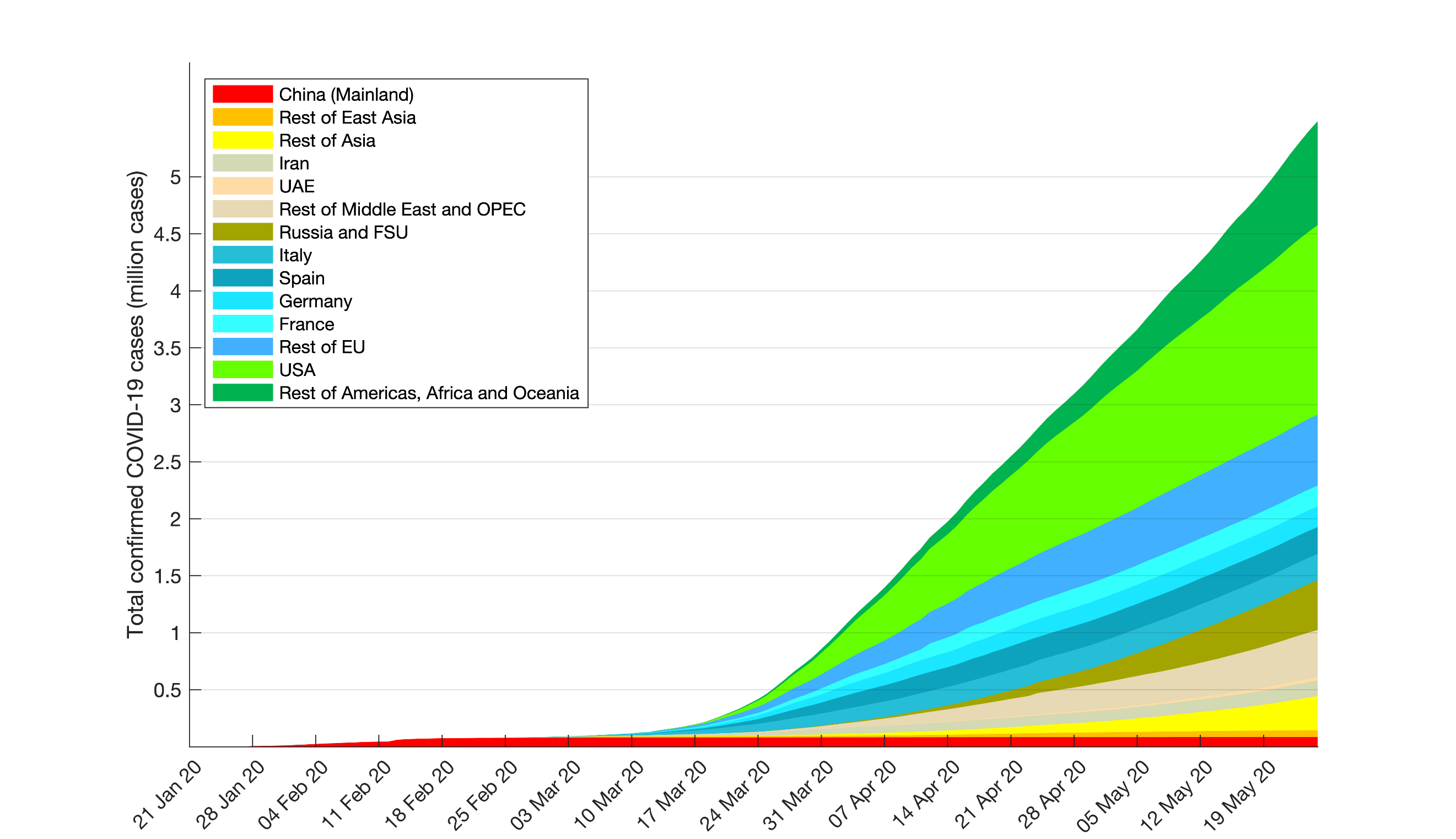


Fig. *SI*1 Graph showing total confirmed COVID-19 cases for 12 aggregated world regions, starting from 21 January 2020 to 25 May 2020.


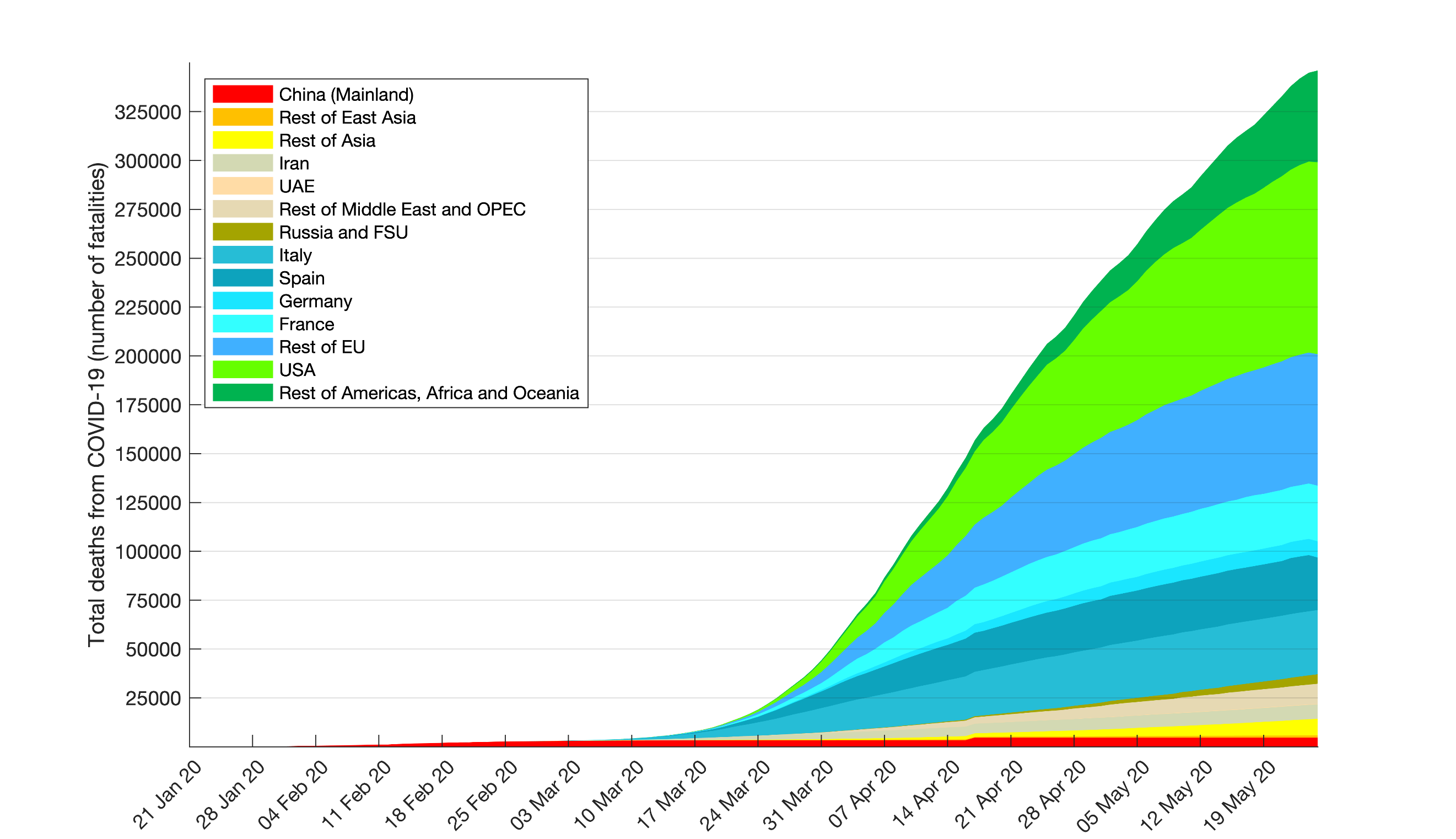


Fig. *SI*2 Graph showing total deaths from COVID-19 for 12 aggregated world regions, starting from 21 January 2020 to 25 May 2020.


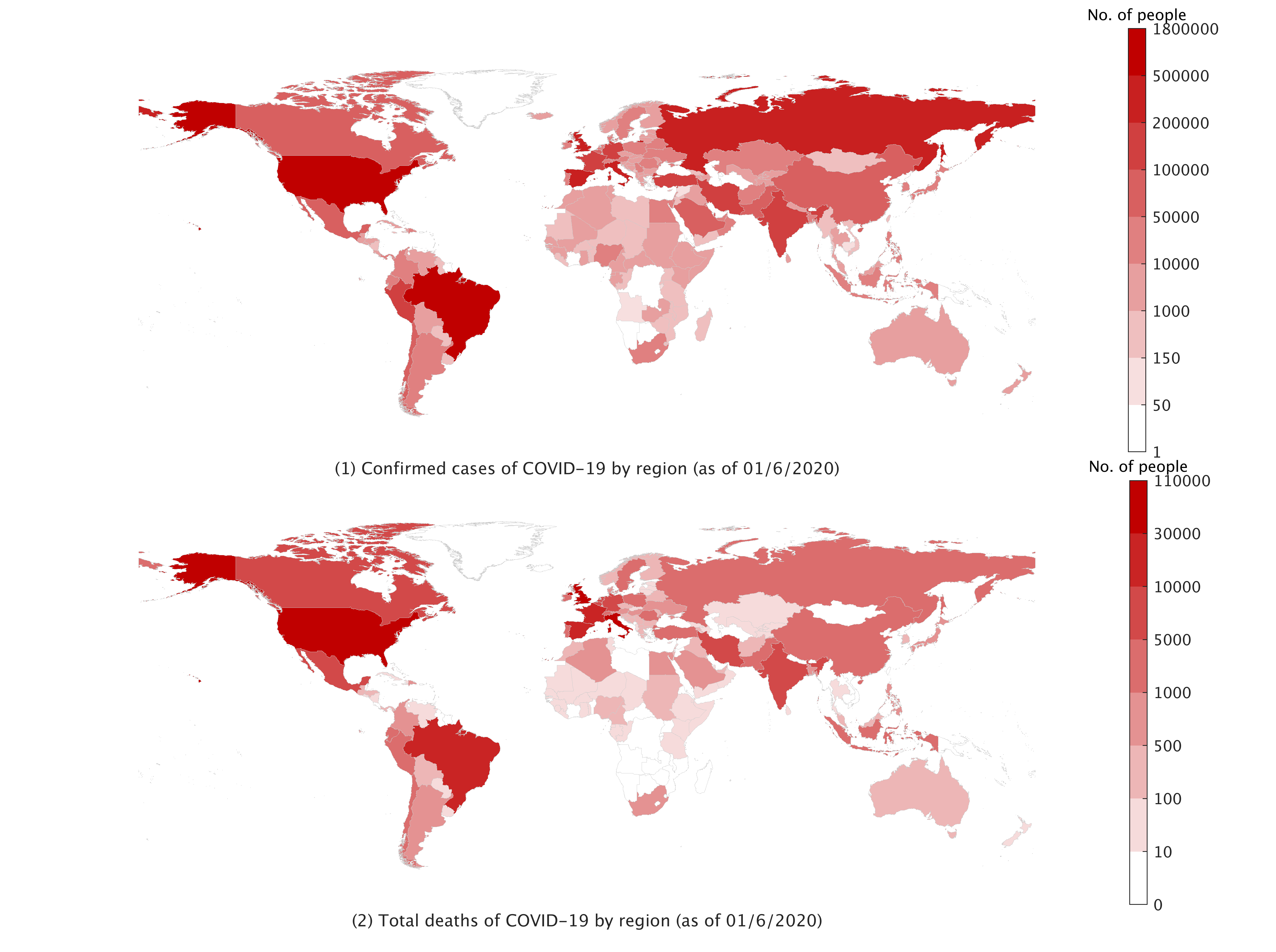


Fig. *SI*3 Graph showing total confirmed cases of COVID-19 and total deaths from the disease for about 210 countries and regions, as of 1 June 2020. Data taken from the Worldometer database ^1^.

# **SI 2 – MRIO compilation, and regional and sectoral classifications**

## SI 2.1 MRIO database compilation in Virtual Labs

The multi-regional input-output (MRIO) tables used for this project were generated using the Global MRIOLab suite^2,3^. The Global MRIOLab allows for the construction of purpose-built global MRIO tables. The Global MRIOLab suite features a raw data repository containing raw data from a large number of data providers. These include national data obtained from various national statistical agencies (such as National Accounts data), data from multi-national agencies such as Eurostat, international trade data such as UN Comtrade and other global data sources, mainly provided by the United Nations Statistics Division (such as the UN System of National Accounts Main Aggregates and Official Country data).

When building an MRIO table, the Global MRIOLab allows the user to

- customise the regional resolution,
- customise the sectoral resolution (both heterogeneous and homogeneous sector structures are supported), and
- choose the individual source data sets that should be included in the MRIO building process.

The raw data are then converted into the regional and sector classification as chosen by the user, and act as the set of external data during the compilation process of the MRIO. The compilation process of any MRIO in the Global MRIOLab can be summarised in the following steps:

1. **Generating an initial estimate.** The initial estimate is a first approximation of the MRIO. The Global MRIOLab offers a number of different routines to generate this initial data set. For this study, an initial estimate routine based on the Eora initial estimate routine was employed^4,5^.
2. **Vectorisation of the MRIO.** This is a preparatory step for constraint formulation.
3. **Formulation of the constraints.** Each data point represented in the set of external data is translated into a linear mathematical constraint. Due to the vectorisation performed in the previous step, all constraints can be stacked into a constraint matrix^6^.
4. **Solving the optimisation problem.** Solving the optimisation problem achieves the reconciliation of the initial estimate MRIO table subject to the constraints defined by the external data. The resulting MRIO ideally reflects the information provided by the external data sets^7^.

By utilizing a constrained optimization approach, the Global MRIOLab is able to reconcile disparate and conflicting external data into a single harmonised MRIO. Hence, MRIO tables generated using the Global MRIOLab can represent information from potentially contradictive data sources, therefore allowing for vast amounts of information to be reflected in the final table. Additionally, MRIO tables generated with the Global MRIOLab include reliability data for each data point of the MRIO^4,6^.

The same approach is followed when constructing satellite blocks.

## SI 2.2 Regional detail used in the MRIO table of this study

The MRIO created for this study depicts the following regions:

| 1 | China (Mainland) |
| --- | --- |
| 2 | South Korea |
| 3 | Japan |
| 4 | Singapore |
| 5 | Hong Kong |
| 6 | Taiwan |
| 7 | Rest of East Asia |
| 8 | Thailand |
| 9 | Indonesia |
| 10 | Malaysia |
| 11 | Rest of South East Asia |
| 12 | India |
| 13 | Rest of South Asia |
| 14 | Australia |
| 15 | Rest of Oceania |
| 16 | Russia |
| 17 | Rest of FSU |
| 18 | Iran |
| 19 | UAE |
| 20 | Middle East |
| 21 | Rest of OPEC |
| 22 | Nigeria |
| 23 | South Africa |
| 24 | Rest of Africa |
| 25 | Italy |
| 26 | France |
| 27 | Germany |
| 28 | Spain |
| 29 | UK |
| 30 | Scandinavia |
| 31 | Rest of EU |
| 32 | Rest of Europe |
| 33 | USA |
| 34 | Canada |
| 35 | Mexico |
| 36 | Rest of Central America |
| 37 | Brazil |
| 38 | Rest of South America |

The rationale for the choice of the regional classification is provided in *SI* 4. Detailed definitions of the individual country/region make-up of these regions can be read from the regional aggregator (sheet RegAgg in the enclosed Excel data file). This regional aggregator establishes a concordance with the countries and areas included in the United Nations’ National Accounts Main Aggregates database^8^.

##


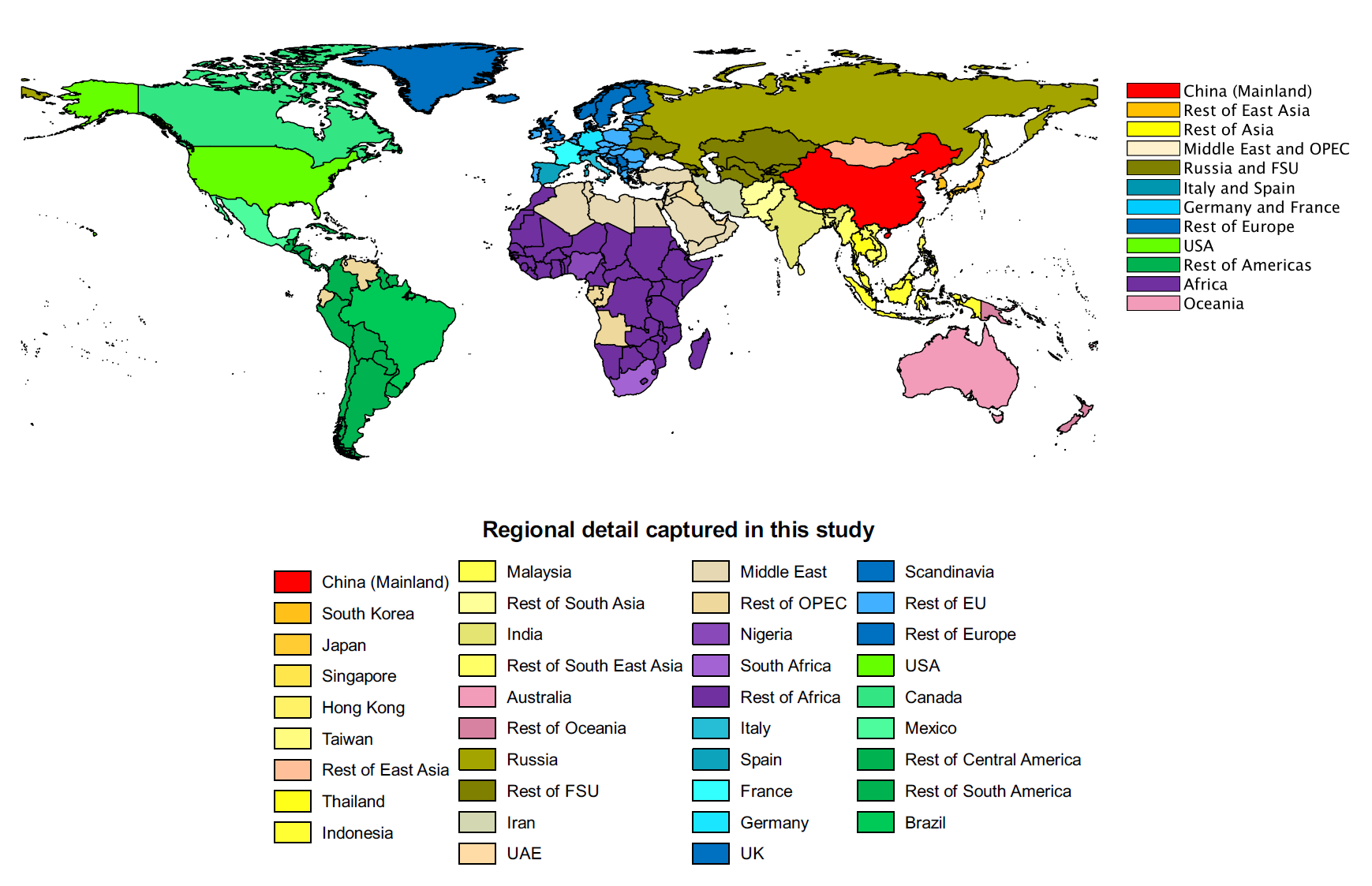


Fig. *SI*4 Regional detail captured in this study, shown as 38 regions. The 38 regions are aggregated into 12 categories, listed in the legend on the right of the figure. *China (Mainland): China (excludes Hong Kong and Taiwan).*

## SI 2.3 Sectoral detail used in the MRIO table of this study

The MRIO created for this study depicts the following sectors:

| 1 | Agriculture |
| --- | --- |
| 2 | Forestry, wood, paper |
| 3 | Fishing |
| 4 | Mining |
| 5 | Food products |
| 6 | Solid fuels |
| 7 | Liquid fuels |
| 8 | Gaseous fuels |
| 9 | Chemicals & plastics |
| 10 | Textiles & leather products |
| 11 | Metal products |
| 13 | Equipment |
| 12 | Ceramic & other manufacturing |
| 14 | Construction |
| 15 | Electricity generation |
| 16 | Gas supply |
| 17 | Air transport |
| 18 | Other transport & storage |
| 19 | Tourism & hospitality |
| 20 | Retail & wholesale trade |
| 21 | Education |
| 22 | Finance & insurance |
| 23 | Business services |
| 24 | Public services |
| 25 | Health care |
| 26 | Private services |

The rationale for the choice of the sectoral classification includes

- isolating hard-hit sectors (air transport, tourism);
- delineating sectors affected by lockdowns (e.g. entertainment and private services);
- singling out health care, chemical products and food, because these may experience increased supply; and
- distinguishing important individual commodities in the supply chains of sectors above, such as refinery fuels, coal, crude oil, and natural gas, and agriculture.

Detailed definitions of these sectors can be read from the sectoral aggregator (sheet SecAgg in the enclosed Excel data file). This sectoral aggregator establishes a concordance with the United Nations’ Harmonised System classification^9^.

# **SI 3 – MRIO and satellite accounts: data sources and non-linear optimization problem**

## SI 3.1 Regional detail used in the MRIO table of this study

The monetary MRIO table was constructed in the Global MRIO Lab^2^ using the following data sources:

1. United Nations Main Aggregates Database^8^,
2. United Nations National Accounts Official Data^10^,
3. UNIDO Industrial Statistics database^11^,
4. United Nations Comtrade database^12^,
5. United Nations ServicesTrade database^13^,
6. numerous national input-output tables^14^.

These data are available up to the year 2018. To simulate the world economy for the year 2020, we used country-specific GDP growth rates^15^ to project the 2018 global MRIO table to 2020, and then re-balanced using the KRAS method^6^.

The satellite accounts are constructed using the following data sources:

1. Income is taken from the monetary tables constructed in the Global MRIO Lab^2^
2. Employment is sourced from data published by the International Labor Organization (ILO) for the year 2019^16,17^
3. Global greenhouse gas emissions (including CO_2_ (*GWP*=1), CH_4_ (*GWP*=28), N_2_O (*GWP*=265), CFCs (*GWP*=8925), HFCs (*GWP*=3772), SF_6_ (*GWP*=23500), NF_3_ (*GWP*=16100))^18^, PM_2.5_, SO_2_ and NO_x_ are taken from the most recent EDGAR database (v5.0)^19^. The combined acidification effects of SO_2_ and NO_x_ can be measured in units of SO_2_-equivalents^20^. We report combined SO_2_ and NO_x_ emissions as SO_2_ + 0.7 NO_x_, with 0.7 being the SO_2_-equivalent factor for NO_2_^21^.

## SI 3.2 Uniqueness of the solution of the non-linear optimisation problem

In order for the non-linear optimisation problem to have a unique solution, the Leontief-inverse

$$\mathbf{L} =\left( \mathbf{I}-\mathbf{A} \right)^{-1}$$

must exist.

Given that we are analysing the post-disaster structure of a productive and profitable economy, the following holds for direct requirements matrix $\mathbf{A}$.

- For all elements $a_{ij}$of $\mathbf{A}\in\mathbb{R}^{nxn}$ the inequality $0\leq a_{ij}<1$ holds.
- For each column the inequality

$$\sum_{i=1}^{n} a_{ij}<0$$

holds. These are the *column sums* of $\mathbf{A}$.

We will use the following intermediate result to show that $\mathbf{L}$ exists and is positive definite. Suppose the following holds for row sums (as opposed to the column sums mentioned further up) of $\mathbf{A}$.

$$\sum_{j=1}^{n} a_{ij}<0$$

Then, using the vector

$\mathbf{x}=\left( \begin{matrix} \begin{matrix} 1 \\ 1 \end{matrix} \\ \begin{matrix} 1 \\ 1 \end{matrix} \\ \begin{matrix} \vdots\\ 1 \end{matrix} \end{matrix} \right)\in\mathbb{R}^{n}$,

the equation $\mathbf{Ax}$ gives the row sums of $\mathbf{A}$. Further, we obtain that $\mathbf{Ax}\boldsymbol{<}\mathbf{x}$**,** while at the same time $\left( \mathbf{Ax} \right)_{i}\boldsymbol{>}0$, for each $i$ because every sector is productive. Hence, we obtain that

$$\left( \mathbf{I}-\mathbf{A} \right)\mathbf{x}=\mathbf{x}-\mathbf{Ax}>\boldsymbol{0}$$

For all elements of the resulting vector. In fact, this result also holds element-wise. This shows that $\left( \mathbf{I}-\mathbf{A} \right)^{-1}$ exists and is non-negative, which also shows that the requirement

$\mathbf{x}^{T}\left( \mathbf{I}-\mathbf{A} \right)^{-1}\mathbf{x}>0$ for $\mathbf{x}>0$

for positive definiteness is fulfilled.

In order to show that the same holds when the column sum of $\mathbf{A}$ are smaller than 1, we use the following result and conclude that $\left( \mathbf{I-}A^{T}A^{T} \right)^{-1}$ exists and is non-negative. Since $\mathbf{I}$ is symmetric, we have

$\left( \mathbf{I}-\mathbf{A}^{T} \right)^{-1}=\left( \left( \mathbf{I}-\mathbf{A} \right)^{T} \right)^{-1}=\left( \left( \mathbf{I}-\mathbf{A} \right)^{-1} \right)^{T}$,

and given that the transpose of a non-negative matrix is non-negative, we conclude that $\mathbf{L}=\left( \mathbf{I}-\mathbf{A} \right)^{-1}$ exists and is non-negative.

Further insights can be derived from these results, which link the mathematical theory of this section back to the economic world. For example, using the results shown in this section, one can conclude that an economy is only productive if every sector is profitable. This so-called Hawkins-Simon condition is a direct conclusion of the existence of Leontief-inverse.

This existence and positive definiteness of the Leontief-inverse is equivalent to the non-linear optimisation routine used in the disaster analysis to have an existing and unique solution.

# **SI 4 – Data for populating the event (Γ) matrix**

This research is being carried out *‘live’,* and as such one of the greatest challenges is to ensure good and consistent coverage of the events since these are one of the key inputs that lead to the results presented in this article.

## SI 4.1 System boundary in space

Given the global nature of the pandemic, sufficiently granular geographical coverage is extremely important. We used three criteria for including individual countries in our data collection:

1. A country features in the top 20 for GDP^22^– thus suggesting that losses in that country are important at both national and global levels
2. A country features in the top 20 for 2018’s tourism share of GDP^15^– thus suggesting that the unprecedented halt to global travel would have an impact both nationally and globally
3. A country features in the top 20 for number of reported cases of SARS-CoV-2^23^ – thus suggesting that the national impacts will be significant regardless of how this country ranks in terms of global GDP and tourism

This resulted in the data collection at sectoral level for 34 individual countries/regions in *SI*2.2. There might seem to be exceptions of countries not *currently* belonging to any of the three categories above. Their inclusion boils down to them falling into category 3 at some point since the start of this research. Further, we cover 184 countries for tourism and air transport losses and 211 countries for lockdown measures.

When some of the countries that we analysed individually were instead clustered in a region within the MRIO model (e.g. Philippines included in Rest of South Asia or Switzerland included in Rest of Europe) we attributed the share of the country to the region based on the weight of its GDP against the cumulative GDP of the region it belongs to.

For each country/region, 26 overall sectors were considered (see *SI*2.3).


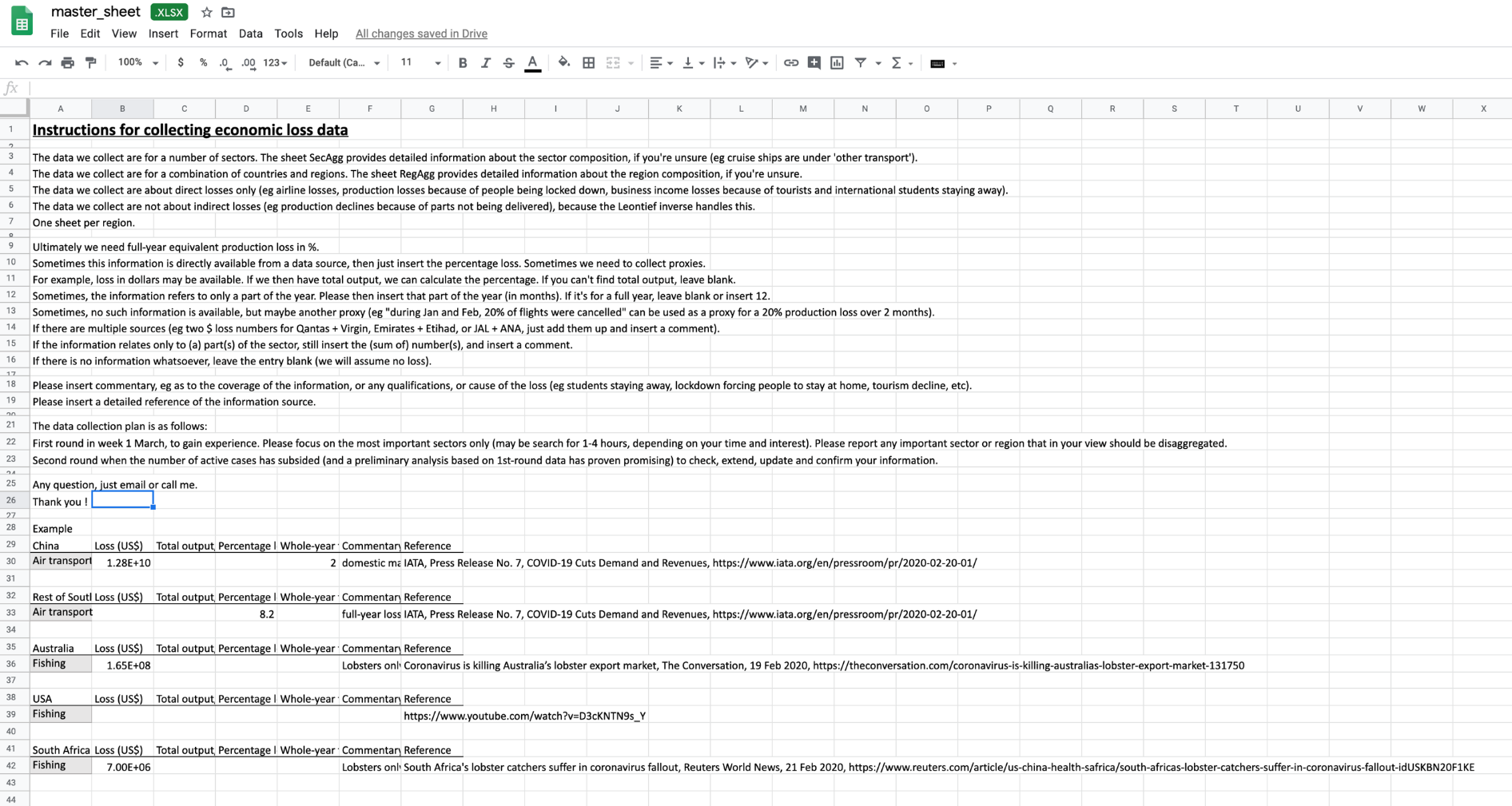


Fig. *SI*5: Data collection template.

| **Country/region** | **Share of global GDP** | **Share of global tourism GDP (2019)** | **Share of SARS-CoV-2 reported cases** |
| --- | --- | --- | --- |
| Australia | 1.67% | 1.69% | 0.14% |
| Austria | 0.53% | 0.59% | 0.32% |
| Brazil | 2.18% | 0.25% | 1.09% |
| Belgium | 0.62% | 1.58% | 5.99% |
| Canada | 2.00% | 1.22% | 1.62% |
| China (Mainland) | 15.86% | 17.86% | 1.62% |
| France | 3.24% | 2.58% | 3.51% |
| Germany | 4.66% | 3.91% | 3.47% |
| Hong Kong | 0.42% | 0.51% | 0.02% |
| India | 3.18% | 2.19% | 2.41% |
| Indonesia | 1.21% | 0.72% | 0.40% |
| Iran | 0.53% | 0.36% | 2.54% |
| Israel | 0.43% | 0.25% | 0.32% |
| Italy | 2.42% | 2.93% | 4.42% |
| Japan | 5.79% | 4.05% | 0.32% |
| Malaysia | 0.41% | 0.47% | 0.14% |
| Mexico | 1.43% | 2.20% | 1.15% |
| Netherlands | 1.06% | 0.57% | 0.87% |
| Nigeria | 0.46% | 0.20% | 0.14% |
| Philippines | 0.39% | 1.02% | 0.26% |
| Portugal | 0.28% | 0.44% | 0.58% |
| Russia | 1.93% | 0.95% | 6.30% |
| Saudi Arabia | 0.91% | 0.82% | 1.31% |
| Singapore | 0.42% | 0.46% | 0.59% |
| South Africa | 0.43% | 0.36% | 0.39% |
| South Korea | 1.89% | 0.53% | 0.22% |
| Spain | 1.66% | 2.23% | 4.53% |
| Switzerland | 0.82% | 0.60% | 0.59% |
| Taiwan | 0.67% | 0.42% | 0.01% |
| Thailand | 0.59% | 1.21% | 0.06% |
| Turkey | 0.89% | 0.96% | 2.98% |
| United Arab Emirates | 0.48% | 0.55% | 0.54% |
| United Kingdom | 3.29% | 2.86% | 4.93% |
| United States | 23.88% | 20.72% | 30.83% |
| Total | 86.64% | 78.24% | 84.61% |

Tab. *SI*4.1 Shares of global GDP, tourism GDP and SARS-Cov-2 reported cases for selected countries/regions (shares for last column valid as of 22^th^ May 2020)

## SI 4.2 System boundary in time

As we mentioned at the beginning of this section, our attempt has been to cover events ‘live’ and incorporate them into the **Γ** matrix – which makes our time-related system boundary somewhat constantly evolving. This differentiates our approach from most of the existing attempts to provide estimates of the economic impacts of SARS-CoV-2 which are based on scenario analyses and/or projections. A notable example is the recently released World Bank East Asia and Pacific Economic Update^24^, which analyses two scenarios (global pandemic and amplified global pandemic) caused by *assuming* four sets of shocks: i) drop in employment by 3%, ii) increase in the international trade costs of imports and exports by 25%, iii) drop in international tourism (captured via a 50 percent tax on inbound and outbound tourist-related services such as transport, accommodation, etc.), and iv) drop by 15% for sectors requiring human interaction. The global pandemic scenario implements the four shocks to the full extent in China while the amplified global pandemic scenario extends these to all countries. Both scenarios produce results in terms of annual percentage reduction to national or macro-regional GDPs, which are obtained through Envisage model calibrated to GTAP Version 10A^25^, a relatively standard computable general equilibrium (CGE) model.

In our case, the time-related system boundary is chiefly limited to the events that *have occurred* (e.g. reported sectoral losses and tourism) or that *have been estimated* to occur (e.g. global impacts on air travel). This translates into economic losses in our model only spanning fractions of a year depending on the country and the stage it is at in the COVID-19 outbreaks, which get updated daily as time goes by – with the sole exception of air transport. This is because neither the International Air Transport Association (IATA) nor the International Civil Aviation Organization (ICAO, an UN-affiliated agency) provide revenue losses for Q1 2020 when this manuscript was prepared. Even though weekly air transport capacities are available via companies specialised in travel analytics^26^, this type of information may not have a global coverage and would require further assumptions to derive revenue losses for the air transport capacity data. As a result, a 12-month forecast by IATA was the most reliable source we could currently locate.

In this analysis, the aim is to estimate the economic losses and environmental gains due to demand reduction with respect to SARS-CoV-2. This includes the reduced consumption associated with preventive (avoidance) behaviors by citizens and the reduced production by firms due to the border control and lockdown policies. Stimulus measures provided by governments, such as interest-free loan, tax relief and cash subsidies to the unemployed, are not included in the analysis. These financial supports generally do not discriminate by sectors. It is impossible for us to allocate these payments to sectors with a reasonable accuracy.

The data streams used in estimating losses are extensively presented in section *SI* 4.4.

## SI 4.3 Data collection procedure and data quality indicators (DQIs)

A three-tiered approach was therefore developed as follows in the attempt to best capture global events related to the SARS-CoV-2.

1. Co-authors were assigned one or more country for which they are responsible. To maximise the opportunity to retrieve as much as possible information also available in languages other than English, co-authors have been allocated countries linked to their language skills and familiarity with the country/region. This allowed us to utilise sources in eleven other languages. In addition to English, and in alphabetical order, Arabic, Chinese, Farsi, French, German, Hindi, Indonesian, Italian, Japanese, Portuguese and Spanish were used in our search, thus covering languages spoken by over 5 billion people.
2. Collected data by co-authors is sent to two co-ordinators who in turn check every single data entry to ensure information in the original source is correctly interpreted and as free from misinterpretation as possible. When errors and inaccuracies are spotted, or doubts arise, the data is returned to the co-author who collected it along with a guide on how to fix it.
3. Lastly, before each run of the model an overall check of the **Γ** matrix is carried out.

Given the recency of the pandemic, a large number of sources formed part of the secondary data used to populate entries in the **Γ** matrix. To qualify the quality of our data inputs we developed a scale of four data quality indicators (DQI) based on the reliability of the source. Each entry in the **Γ** matrix is scored against the DQIs. A synopsis of the DQIs and the share of different sources used is given in the following table.

| **Data quality indicator** | **Source Description** | **Total entries** | **Share [%]** | **Weighted DQI Average** |
| --- | --- | --- | --- | --- |
| 1 | Peer-reviewed scholarly work, official national (e.g. Governments, trade bodies) or supra-national (e.g. OECD, UN, IATA) | 186 | 52.54% | 1.64 |
| 2 | Global research clusters such as academic consortia, data hubs, or consulting firms | 111 | 31.36% |  |
| 3 | Well-respected national and international newspapers | 55 | 15.54% |  |
| 4 | Others | 2 | 0.56% |  |
|  |  | 354 | 100.00% |  |

Tab. *SI*4.2 Data collection sources

## SI 4.4 Data streams for the Γ matrix

Two main streams of data were used to construct the **Γ** matrix.

The first stream involves sourcing information from secondary data to identify *monetary revenue losses* [in USD], *shares of revenue losses* [as percentage of an overall sector], *whole-year fraction* [as a temporal measure over which the loss occurs], *sector* [out of the 26 sectors above] and of course, the *country/region*. These parameters allow us to estimate the absolute revenue losses by sector for each of the countries and regions considered. This specific sectoral estimate covers cases arising from the pandemic that made into the news such as Italian fashion firms or Australian lobster market. This data stream came entirely from co-authors and was entirely handled through the three-tier process explained above.

A second stream accounts for policy measures in response to SARS-CoV-2 which have limited people’s movement internationally and domestically and imposed various degrees of closures up to national lockdowns. This creates three main effects: reducing the volume of tourism activity, reducing consumption of non-essential goods and services, and reducing private transport. While we include the first two, we exclude the latter in our study. To evaluate those two effects, in the second stream of data, we centralised the data collection and calculation processes (i.e. this was carried out by the coordinators) to generate a coherent, comprehensive and up to date impact on tourism and national lockdowns, respectively.

For tourism, this is not a clearly defined discrete economic sector and its own activity encompasses visitor expenditure on transport, accommodation, food, recreation and shopping^27^. Because of its composite nature, many secondary sources are not able to report a complete tourism revenue loss figure but rather focus on a subcategory (e.g., closing of theme parks) or a particular market segment (e.g., the loss of Chinese visitor). As a result, we adopted the following procedures to systematically capture the reduction of tourism revenues.

*Air transport*. We first rely on International Air Transport Association (IATA) for their assessment on industry revenue losses by country and by major regions. IATA is the trade association for the world’s airlines, representing 290 airlines or 82% of total air traffic and is able to predict revenue losses based on real-time booking and forward reservations. IATA has published four assessments on the SARS-CoV-2 impact on air transport^28^. In this model, we adopt the fourth impact assessment (published on April 14^th^) which captured the situation of air operation in which markets with severe travel restrictions have covered 98% of global passenger revenues^29^.

*Other tourism expenditure*. To estimate losses of other tourism items, we turn to the World Travel and Tourism Council (WTTC) for their annual report on inbound tourism and domestic tourism expenditure for 184 countries. Our estimation procedure first differentiated travel restriction policies that are currently in place for the countries and regions considered, including partial border closure, national border closure, state border closure, and close of non-essential businesses (lock-down). For countries that suspended international flights and closed their national borders, inbound tourism expenditure is assumed to be zero; for those that embrace state level border closure, domestic travel ban, or a full lock down, domestic tourism expenditure is also reduced to none. These government measures were implemented by individual countries progressively at different stages, indicating that individual destinations experience different levels of tourism losses. To estimate these, we first compiled and cross-checked such measures and their implementation date for 184 countries from international and private agencies that systematically report SARS-CoV-2 measures. These include ACAPS, IATA, United Nations Economic Commission for Europe (UNECE), WorldAware, Garda World, and Al Jazeera^30-35^ . Based on this list, a time share was calculated between the date the restrictions policies were implemented and May 22^nd^ (the time the revised manuscript was prepared) for domestic travel and inbound travel respectively. These time shares were then used to calculate the amount of tourism losses by multiplying it by the 2019 WTTC annual tourism expenditure. For countries that still maintain some tourism activities without significant movement restrictions, the level of tourism reduction was estimated using published sources. For example, China Tourism Academy reported that China had experienced 69% tourism level losses for the first quarter in 2020^36^. This information was used to proxy revenue losses for all tourism expenditure in China from Feb 1 to May 22. Once the overall tourism revenue loss for a given country was determined, it was allocated to “hospitality”, “other transport” and “retailing” sectors using tourism satellite account ratios reported from Lenzen et al.^37^.

*Lockdown*. Lockdown data are sourced from the Oxford COVID-19 Government Response Tracker (OxCGRT), a tool that reveals the full extent of restrictions placed on the world population ^38^. The OxCGRT initiated and maintains a global dataset of containment and mitigation measures implemented in every country in the world. This includes a Stringency Index (ranging from 0 to 100) available for each country in the dataset. For this research we assume a lockdown situation to initiate when the Stringency Index rises over 70 and for it to cease when it drops below 70. By tracking the fluctuations of the Stringency Index we have been able to identify begin and end (or current) dates for lockdown for all countries in our analysis. We also cover US states individually by retrieving information from each State’s governor in official publications. While a Stringency Index over 70 might mean slightly different scenarios in different countries we tested the robustness of this specific threshold by cross-checking the dates obtained with dates actually matching lockdown measures being implemented as advertised in global news. For instance, in Italy the first strict lockdown measures were announced on Mar 3^rd^ and the Stringency Index exceeds the 70 threshold the following day on Mar, 4^th^ with a value of 71.67. Similarly, measures began to be eased in Italy on May, 4^th^ and that is also the date when we first observe the Stringency Index for Italy dropping below 70 (65.24).

A lockdown situation for a country gets implemented in the **Γ** matrix for the three main sectors affected: Retail, Education, and Private Services, which are assigned a 100% loss across the duration of the lockdown apart from Retail where the shares linked to food and essential items was subtracted. The 100% loss in Education requires some further specification. It might seem, in fact, that Universities are still receiving the fees due by students currently enrolled. However, there is mounting and sufficient evidence to safely assume that the education sector globally will be severely hit by the pandemic^39^. This is true for both schools^40^ and Universities^41^ and applicable in English-speaking countries that welcome every year high numbers of international students as well as non-English speaking countries, such as Italy^41^ , where the lockdown has seen students entitled to refunds. A detailed estimate of the economic impacts that the lockdowns will entail is however very hard since they might materialise on next year’s enrolment numbers or through a centralised approach in a certain country where all Universities decide to issue full or partial refunds of students’ fees^42^. Some more immediate impacts that have materialised already are linked to student accommodation costs, where students have been allowed to leave their accommodation immediately and waived any remainder that was due^43^. Also, the closure of thousands of University campuses globally has implications upstream in the supply chain that would not be captured unless a sectoral loss is attributed to Education. Lastly, the Education sector has started to experience losses long before national lockdowns began. This is particularly true for language schools, instantly hit by the initial travel concerns^44^, as well as all other schools where parents started to keep kids home due to coronavirus concerns ^45^. To capture this mix of events and impacts, we therefore believe that a 100% loss limited solely to the lockdown period to date represents a conservative hypothesis with respect to the actual losses that the Education sector will experience globally and that already started populating global news^46,47^.

Retail, Education, and Private Services are broad sectors and therefore some allocations of loss may cover sub-sectors that are not affected by any losses (e.g. waste collection included in *Private Services*) and in other instances we have not allocated losses where there were some in reality (e.g. child day care not included in *Healthcare*). Such in- and exclusions are likely to sway results in opposite directions, and ultimately lead to uncertainties in the sectoral breakdowns of our results. More generally, such circumstances point to a fundamental problem in economic analyses, such as CGE modelling and all input-output modelling: sector aggregation. Whilst sector aggregation can in principle be overcome by disaggregating the databases underlying the modelling exercise, there are limits to how far this can be taken. These limits are primarily posed by the availability of information, and this is especially true for global analyses that include developing countries. They are to a lesser extent posed by limitations of computer memory. The largest MRIO databases to date distinguish about 15,000 region-sector pairs^2^, and such databases require significant memory and runtime to be handled during compilation and matrix inversion operations.

Notwithstanding the limitations above, we constructed the **Γ** matrix based on our current understanding of how long the travel restriction is, what type of containment and mitigation measures are and have been implemented, and the extent of damages to different sectors where this information is available from a reliable estimate. For example, we know more about the losses to air transport, the losses of tourism, and the effect on retailing, education and private services due to nation-wide lockdowns, than other sectors (although the ripple effect on these sectors from losses in Retail does get captured by the IO formulation). We are also cautious on how changes of government measures may influence our estimations in the G matrix. From June 2020, countries began to lift mobility restrictions for domestic travel and some for international journeys, for example, the USA (the end of May by states), Australia (June 5^th^), New Zealand (June 8^th^), and European Union (June 15^th^). This allows travel to resume and every day operations to gradually get back to “normal”. Therefore, we chose to estimate the economic losses from the start of the pandemic to the end of May when countries were still in the lockdown phases. We have assembled the best current knowledge and included projections in the only instance where we can strongly justify it (IATA 2020 air travel) and in lieu of any better source of information. Therefore, we believe our model is likely to produce results closer to the lower bound of the real impacts which can only be determined *ex-post*.

# **SI 5 – Limitations, uncertainty and sensitivity analysis**

This work is concerned with determining estimates for reduced post-disaster consumption possibilities in the wake of the COVID-19 pandemic. As such, we use the disaster analysis method by Steenge and Bočkarjova^48^, and apply to a global setting described by a global MRIO database^2^. There are limitations and uncertainties involved with using any (MR)IO method; in our case these are four-fold: i) there are three variants of method by Steenge and Bočkarjova^48^ (*SI* 5.1), ii) MRIO and loss-data carry data uncertainty (*SI* 5.2), iii) underreporting of losses and uncertainty about future developments can introduce systematic errors (*SI* 5.3), and iv) as with all IO analyses, certain simplifying assumptions are made about the functioning of the economy (*SI* 5.5). In the following we will deal with these four types of limitations.

Further, we will investigate the robustness of our results (*SI* 5.4), by undertaking three sensitivity analyses. In these, we examine the sensitivity of our results under a variation of the MRIO table compilation process, a systematic variation of the entries in the **Γ** matrix for air transport, and variations of all entries in the **Γ** matrix.

## SI 5.1 Method uncertainty

Applying the original disaster analysis method by Steenge and Bočkarjova^48^ can lead to solutions featuring negative consumption possibilities. In a regional setting, this can be interpreted as the sectors in that region requiring assistance from outside the region in order to uphold levels of final demand. However, in a global setting, this is clearly impossible^49^. As a consequence, production reduces further than Steenge and Bočkarjova’s post-disaster output $\tilde{\mathbf{x}}\boldsymbol{=}\left( \mathbf{I}\boldsymbol{-}\boldsymbol{\Gamma} \right) \mathbf{x}$, where **x** is pre-disaster output, and **Γ** is Steenge and Bočkarjova’s event matrix, where diagonal elements *Γ_ii_* describe the relative loss of industries *i*=1,…,*N* as a direct result of a disaster.

This further reduction has been modelled in two ways. First, in their application to severe space weather events, Schulte in den Bäumen *et al.*^50^ exploit the fact that many of the production inputs in the input coefficients (**A**) matrix have such small values that they can be considered to occupy a marginal, or non-essential role for production. In other words, production is assumed to be able to continue without these inputs. An example is catering for fairs and expos held by the car manufacturing industry. These production inputs can be excluded from the **A** matrix, by setting a threshold *τ*, and setting $A_{ij}=0 \forall A_{ij}<\tau$. Then, Steenge and Bočkarjova’s formalism is repeated, until all negative final demand disappears. A range of thresholds *t* can be applied. This method has also been employed in a study on the effects of major floods in Germany^51^, and to an assessment of the spill-over effects of Australian tropical cyclone Debbie^52^.

Second, in their application to earthquakes and typhoons affecting Taiwan, Faturay *et al*.^53^ estimate optimal consumption possibilities, by maximising post-disaster output $\tilde{\mathbf{x}}$, subject to two conditions: First, they ask that $\tilde{\mathbf{x}}\boldsymbol{\leq}\left( \mathbf{I}\boldsymbol{-}\boldsymbol{\Gamma} \right) \mathbf{x}$, where **x** is pre-disaster output. Second, they require that post-disaster final demand $\tilde{\mathbf{y}}\mathbf{=}\left( \mathbf{I-A} \right) \tilde{\mathbf{x}}\boldsymbol{\geq}0$ is strictly non-negative. The solution $\tilde{\mathbf{y}}$ of this optimisation problem are the post-disaster consumption possibilities. This method has also been applied in a study of the effects of climate change on food supply in the State of New South Wales, Australia^54^.

In this work, we employ the method by Faturay *et al*.^53^, because i) it avoids the negative final demand outcomes inherent in the method by Steenge and Bočkarjova^48^, and ii) it arrives at an optimal solution that maximizes post-disaster industrial output, and as such avoids overly mechanistic assumptions about the importance of production inputs as well as the problem of choosing an arbitrary threshold *τ*.

## SI 5.2 Data uncertainty

The same technique was applied for quantifying the uncertainties in the carbon footprint of tourism assessment^55^. Therefore, the text presented below is taken verbatim from Lenzen et al. 2018^55^.

Data taken from primary sources (*SI* 4) are associated with measurement errors. MRIO databases are compiled from these primary data, and therefore, uncertainties propagate from the raw data, via a compiled MRIO, to final economic and environmental impact measures^56-58{Heijungs, 2014 #5983}^. Because IO theory is non-linear, uncertainties of consumption losses and associated impacts cannot be determined analytically, but require Monte-Carlo simulation techniques to be applied ^37,59-61^.

More specifically, we follow previous analyses^62,63^, and propagate uncertainty using standard deviations^64^ $\sigma_{\mathbf{Q}}, \sigma_{\mathbf{T}}$ and $\sigma_{\mathbf{y}}$ (sourced from the Global MRIO Lab ^2^ for perturbing the basic input-output quantities **Q**, **T** and **y**, then calculating perturbed consumption possibilities, and then gathering these for a large number of perturbation runs. Standard deviations of derived economic and environmental impacts are then taken from the statistical distribution of the perturbations. More specifically, we generate normally distributed random numbers $\nu\mathbb{\in N(-}1|1)$ and use these to perturb the basic impact equation (see main text) $F=\mathbf{Q}{\hat{\mathbf{x}}}^{-1}\left( \mathbf{I-}\mathbf{T}{\hat{\mathbf{x}}}^{-1} \right)^{\boldsymbol{-}1}\left( \tilde{\mathbf{y}}\boldsymbol{-}\mathbf{y} \right)$ ^56^ to:

$F^{p}\mathbf{=}\mathbf{Q}^{p}{\hat{\mathbf{x}^{p}}}^{-1}\left( \mathbf{I}-\mathbf{T}^{p}{\hat{\mathbf{x}^{p}}}^{-1} \right)\left( {\tilde{\mathbf{y}}}^{p}\boldsymbol{-}\mathbf{y}^{p} \right)$ , (*SI* 5.1)

where $\mathbf{x}^{p}=\mathbf{T}^{p}\mathbf{1}^{N}+\mathbf{y}^{p}$, and where the logarithmic perturbations

$\mathbf{Q}^{p}={10}^{\log_{10}\mathbf{Q}+\nu\sigma_{\log_{10}\mathbf{Q}}}$, $\mathbf{T}^{p}={10}^{\log_{10}\mathbf{T}+\nu\sigma_{\log_{10}\mathbf{T}}}$, and $\mathbf{y}^{p}={10}^{\log_{10}\mathbf{y}+\nu\sigma_{\log_{10}\mathbf{y}}}$ (*SI* 5.2)

ensure that neither $\mathbf{Q}^{p}$, $\mathbf{T}^{p}$ nor $\mathbf{y}^{p}$ will ever become negative.^56^ Here, the standard deviation of logarithms can be approximated by^64^

$\sigma_{\log_{10}x}\approx\log_{10}\left( x+\sigma_{x} \right)-\log_{10}\left( x \right)=\log_{10}\left( \frac{x+\sigma_{x}}{x} \right)$ . (*SI* 5.3)

We also perturbed post-disaster consumption losses $\tilde{\mathbf{y}}\boldsymbol{-}\mathbf{y}$ according to $\left( \tilde{\mathbf{y}}\boldsymbol{-}\mathbf{y} \right)^{p}={10}^{\log_{10}\left( \tilde{\mathbf{y}}\boldsymbol{-}\mathbf{y} \right)\mathbf{-}\left| \nu\right|\sigma_{\log_{10}\left( \tilde{\mathbf{y}}\boldsymbol{-}\mathbf{y} \right)}}$, however here we used only strictly negative perturbations, assuming that the unperturbed values $\tilde{\mathbf{y}}\boldsymbol{-}\mathbf{y}$ represent a lower bound on actual losses, given that post-disaster consumption $\tilde{\mathbf{y}}$ results out of an optimisation exercise. This feature leads to our probability distributions being asymmetric (Fig. *SI* 6). We assumed $\sigma_{\tilde{\mathbf{y}}\boldsymbol{-}\mathbf{y}}=100\%$ for air transport and tourism (since air transport data are the only data we use that include whole-of-year projections, hence are unlikely to be affected by more than 100% uncertainty), $\sigma_{\tilde{\mathbf{y}}\boldsymbol{-}\mathbf{y}}=300\%$ for all sectors affected by lockdowns (to account for lockdown periods to be extended up to 3-fold; see *SI* 4), and an intermediate value of $\sigma_{\tilde{\mathbf{y}}\boldsymbol{-}\mathbf{y}}=200\%$ for remaining sectors, and proceeded with the log-transformation in Equation *SI* 5.3.

In our Monte-Carlo analysis, perturbations as in equation *SI* 5.1 are repeated *P* times, and outcomes $F^{(p=1,\ldots,P)}$ collected. Finally, a normal distribution is fitted to a frequency plot of the $F^{(p)}$, and the standard deviation $\sigma_{F}$ derived from the fit. In order to obtain an accurate estimate of $\sigma_{\tilde{Q}}$, *P* needs to be large enough for the frequency plot of the $F^{(p)}$ to be smooth. In this work, *P* = 10^4^.

Errors assessed using Monte-Carlo-type approaches are stochastic. This means that a sum of a large number of summands is relatively less uncertain than each summand. Matrix products involving the Leontief inverse represents a sum over a very large number summands, and hence the reduction of relative uncertainty applies here. This explains that despite significant perturbations of the consumption losses $\sigma_{\tilde{\mathbf{y}}\boldsymbol{-}\mathbf{y}}\lesssim100\%$, overall standard deviations are below 10% (Fig. *SI*6). Of course, these do not reflect systematic uncertainties (see *SI* 5.3).


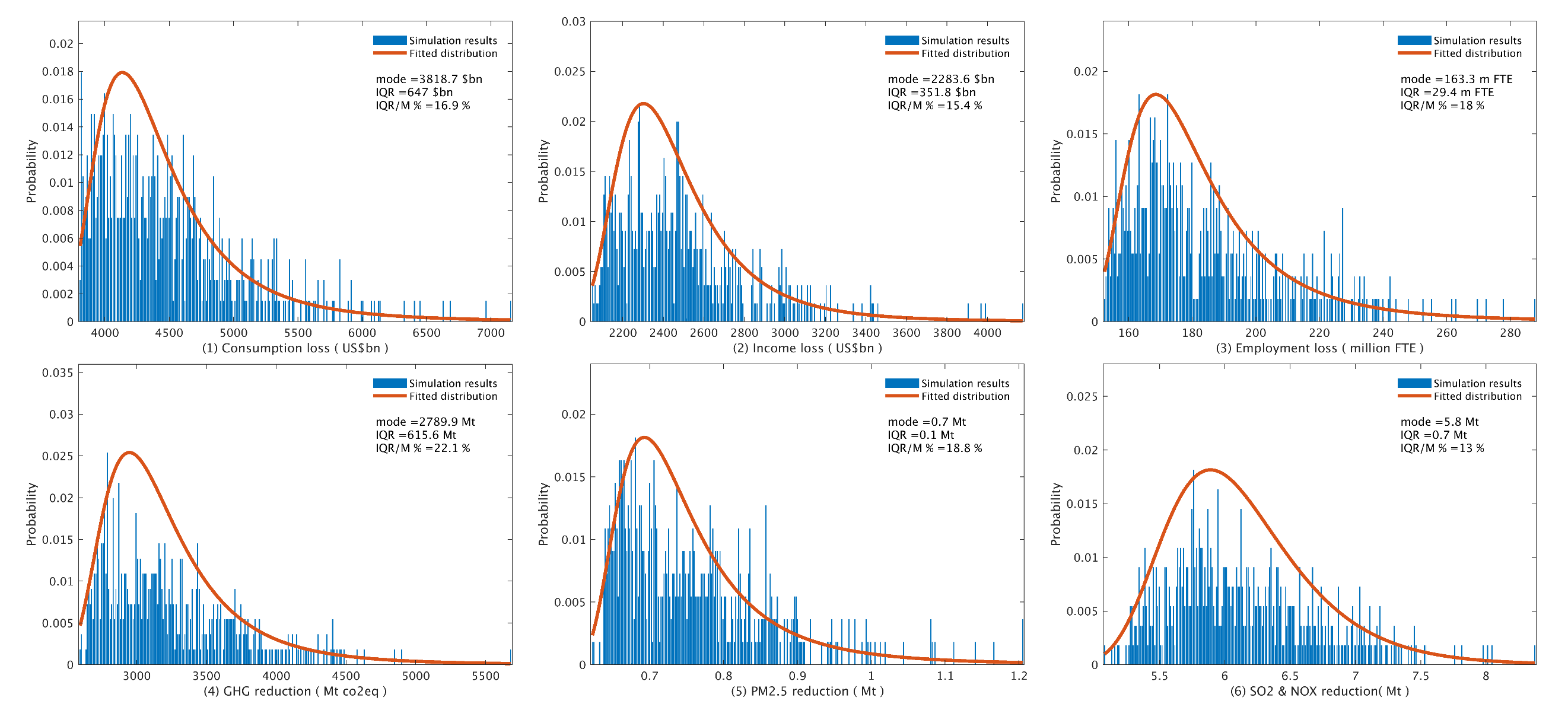


Fig. *SI*6: Probability distributions resulting out of Monte-Carlo simulations of our MRIO disaster analysis. The stochastic standard deviations are derived by fitting a normal distribution.    *Mode*: value that appears most often; *IQR*: interquartile range, being equal to the difference between 75th and 25th percentiles. *IQR/M*: ratio of IQR and mode, measuring the variability.

## SI 5.3 Systematic uncertainty

There exist three sources of systematic (non-stochastic) uncertainty. First, the focus of this study is to provide a timely estimate of the spill-over effects of the 2020 Coronavirus pandemic. At the time of publication, the number of actual cases were declining in some countries. We’re proposing an approach that captures events as they happened and can be updated and upgraded as things unfold. This leads to a potential systematic underestimation of entries in the event matrix $\boldsymbol{\Gamma}$, and in turn of impacts. Also, when sectoral losses were given in percentages it was not always possible to estimate whether the loss referred to the 2019 registered turnover or 2020 projected estimates.

Second, cases of infections^65-68^ as well as monetary losses may have been underreported. Again, this leads to a potential systematic underestimation of entries in the event matrix $\boldsymbol{\Gamma}$, and in turn of impacts.

Third, we have not been able to locate comprehensive quantitative information about increased health care expenditures, and the increases because of grocery panic-buying. As a result, our calculations do not include all potential increases of emissions and employment, for example. This leads to a potential systematic overestimation of negative impacts.

## SI 5.4 Sensitivity analyses

In order to examine the robustness of our results, we undertake three sensitivity analyses. In these, we examine the sensitivity of our results under i) a variation of the MRIO table compilation process, ii) a systematic variation of the entries in the **Γ** matrix for air transport, and iii) variations of all entries in the **Γ** matrix.

***Variation of MRIO table compilation***

Not one global MRIO matrix is the same as another, because of a multitude of reasons^69,70^. First, different teams use different construction pipelines and algorithms for data reconciliation and harmonisation. Second, different databases use different regional and sectoral classifications. Third, different teams access different sets of raw data to base their construction processes on. These circumstances can lead to different expressions of MRIO tables, however results generally converge in applications^71-74^.

Here we investigate the third issue – different underlying data – because it has been found as the dominant cause for MRIO data divergence^75,76^. More specifically, we use the Global MRIO Lab to run an alternative MRIO data set, that uses the six data sources listed in *SI*3, but excludes the main source, the UN Main Aggregates database amongst the primary data sources. Of course, as with the table used to generate our results, the alternative table still adheres to all accounting and balancing requirements.

|  |  | Main  MRIO table | Alternative  MRIO table | Relative deviation |
| --- | --- | --- | --- | --- |
| Consumption | US$tr | 3.805 | 3.646 | 4.2% |
| Employment | million FTE | 147.16 | 142.91 | 2.9% |
| Income | US$tr | 2.065 | 1.988 | 3.7% |
| GHG emissions | Gt CO_2_-eq | 2.511 | 2.372 | 5.5% |
| PM2.5 emissions | Mt | 0.632 | 0.609 | 3.6% |
| Air pollutants | Mt | 5.059 | 4.807 | 5.0% |

Tab. *SI*5.1: Summary of deviations in main results from varying the main data source for compiling the underlying MRIO table.

Despite the omission of the MRIO table’s main underlying data source, the key results change by around or less than 5% (Tab. *SI*5.1).

***Systematic variation of the entries in the Γ matrix for air transport***

Our second approach to addressing the sensitivity of reported losses, we systematically changed the ***Γ*** matrix entries for air transport. Air transport links all sectors by moving goods and passengers around, which is important for both final consumption and intermediate inputs for many businesses. At the same time, air transport is carbon intensive, responsible for at least 2% of global carbon emissions^77^. Given the sector’s critical role in the economy, air transport losses are expected to be influential in terms of both economic output and emissions. The effect of this potential sensitivity is investigated via comparing two sets of forecasts provided by IATA: US$252 billion from the third assessment report (referred to as *scenario 1*) and US$ 314 billion from the fourth assessment report (*scenario 2*).

The further grounding of air transport in scenario 2 creates additional ripple effects across the economy, reducing economic losses from 4.02% (scenario 1) to 4.20% (Figure *SI*7). Service, trade, and transport & tourism are the main sectors additionally affected. Scenario 2 leads to a 4.50% emission reduction, higher than the 4.24% reduction in scenario 1. Overall, the sensitivity analysis supports the critical role of air transport in modelling the COVID-19 pandemic event. Emissions reductions are more sensitive than economic losses to variations in direct air transport downturns.


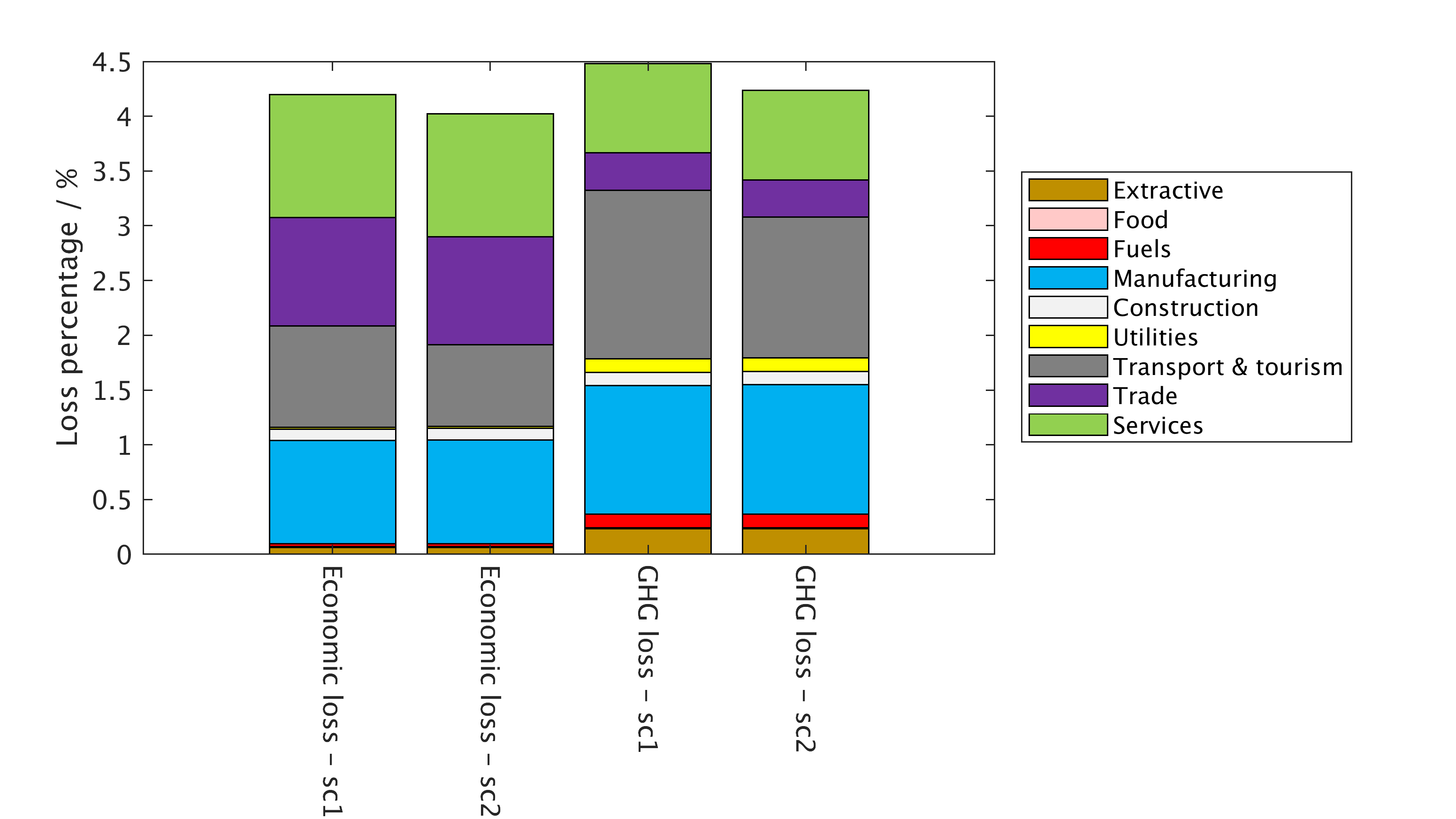


Fig. *SI*7: Economic and emission losses from two sensitivity scenarios. Scenario 1 uses the IATA third assessment report, and Scenario 2 adopts the IATA fourth assessment report, *ceteris paribus*.

***Variations of all entries in the Γ matrix***

Our final analysis involves the perturbation of all entries in the **Γ** matrix. In order to obtain an understanding of the influence of every individual entry *Γ_ij_* in the **Γ** matrix, we perturbed this entry by 25%, and re-ran the disaster analysis and all results. We focus on reported GHG emissions, because these undergo the largest relative reduction as a result of the pandemic (see Tab. *SI*5.1 above).

Our analysis shows that none of the **Γ** matrix is able to individually sway the overall GHG reduction figure by more than 1% (Fig. *SI*8).


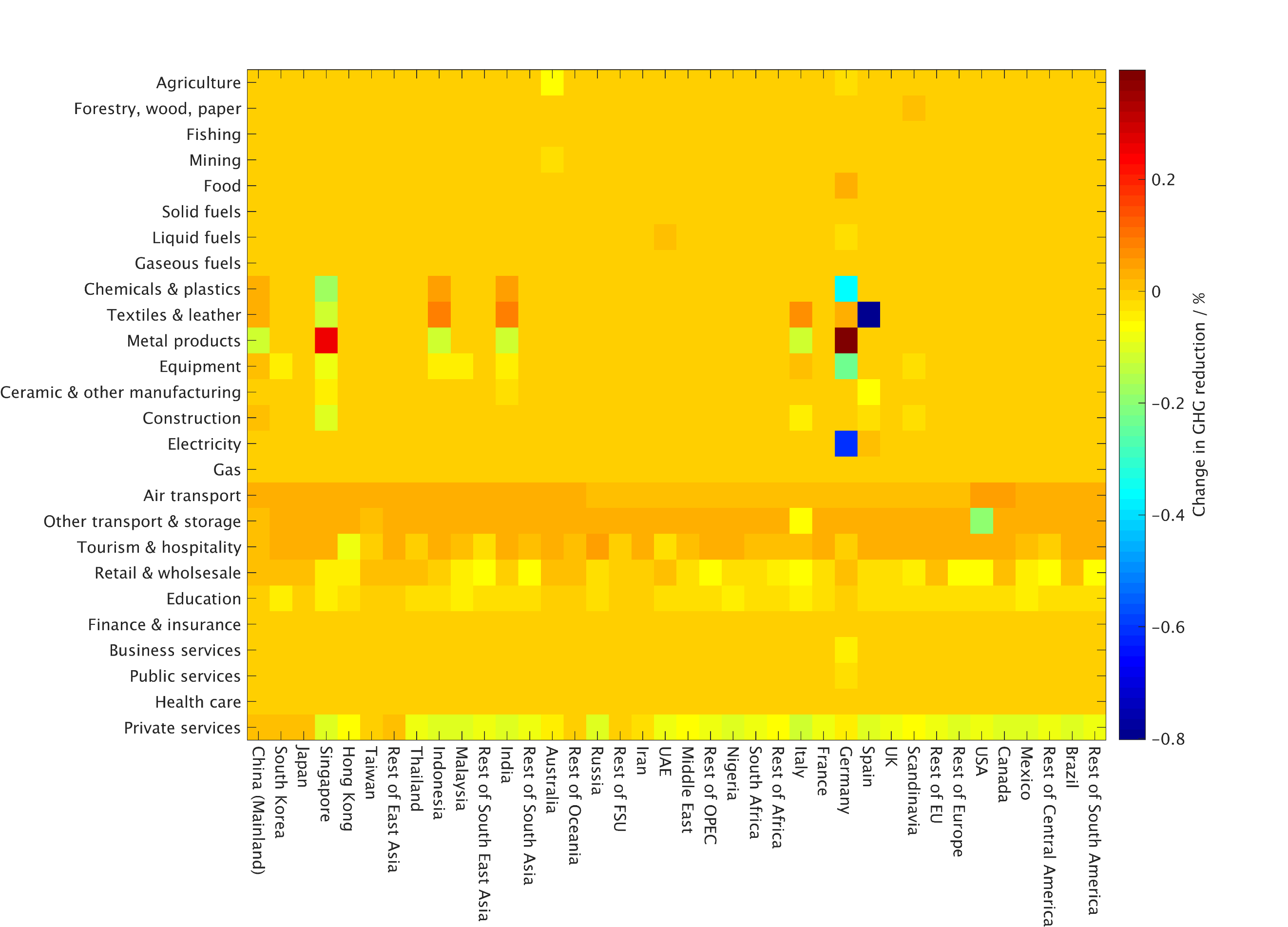


Fig. *SI*8: Sensitivity (in % relative deviation from unperturbed result) of Covid-19-related reductions in global GHG emissions as a result of a 25% perturbation of individual entries in the **Γ** matrix.

## SI 5.5 Other limitations

As with every input-output analysis, this study is affected by the usual limitations of the input-output method. First, this analysis is static in that it does not incorporate price effects of shortages, that in turn regulate demand, and eventually lead to a post-disaster equilibrium. However, since the effects of the COVID-19 pandemic are very short-term, and because governments are implementing rent freezes and countermeasures to price gouging, the static-shock approach taken by input-output disaster analysis appears appropriate. Second, we assume a fixed production recipe, i.e. the coefficients of the input-output technology matrix **A** are assumed to be constant. This assumption introduces a certain degree of rigidity into the disaster calculus. In this work, these two issues probably do not pose significant limitations, because our analysis concerns effects that play out over the very short-term (approximately three months), during which there may be little time for price signals to penetrate markets, and and for production input structures to be adjusted.

Second, as with Steenge and Bočkarjova’s method, this work assumes sector-specific homogeneous production shortfalls, i.e. reductions in supply as specified by the events matrix **Γ** are equal across intermediate and final demand categories, and are equal for domestic supply and exports. In reality, especially the latter condition might not hold, as governments seek to protect domestic populations first from perceived shortages of essential goods and services.

# **SI 6 – Additional results**

## SI 6.1 Data table for Fig.1

| Region | Consumption loss (US$bn) | Income loss (US$bn) | Employment loss  (million FTE) | GHG reduction (Mt CO2eq ) | PM2.5 reduction  (Mt) | SO2 & NOx reduction  (Mt) |
| --- | --- | --- | --- | --- | --- | --- |
| China (Mainland) | 490.15 | 248.72 | 22.25 | 473.50 | 0.28 | 1.00 |
| South Korea | 70.65 | 38.60 | 1.57 | 47.60 | 0.01 | 0.09 |
| Japan | 99.14 | 43.54 | 1.69 | 65.38 | 0.01 | 0.12 |
| Singapore | 56.25 | 24.58 | 0.96 | 27.18 | 0.01 | 0.07 |
| Hong Kong | 50.80 | 23.61 | 1.16 | 21.07 | 0.01 | 0.07 |
| Taiwan | 37.13 | 18.40 | 1.03 | 33.32 | 0.01 | 0.10 |
| Rest of East Asia | 3.37 | 1.23 | 1.53 | 18.22 | 0.01 | 0.07 |
| Thailand | 31.31 | 10.04 | 2.52 | 30.99 | 0.01 | 0.07 |
| Indonesia | 39.17 | 14.40 | 5.28 | 80.58 | 0.02 | 0.32 |
| Malaysia | 48.78 | 22.35 | 2.32 | 47.42 | 0.01 | 0.11 |
| Rest of South East Asia | 68.98 | 26.45 | 11.12 | 79.17 | 0.02 | 0.17 |
| India | 77.41 | 41.51 | 8.89 | 70.98 | 0.03 | 0.14 |
| Rest of South Asia | 20.91 | 10.28 | 7.50 | 42.34 | 0.00 | 0.05 |
| Australia | 93.30 | 50.80 | 1.24 | 84.69 | 0.01 | 0.17 |
| Rest of Oceania | 24.50 | 10.40 | 0.51 | 18.31 | 0.00 | 0.05 |
| Russia | 58.53 | 87.33 | 9.81 | 81.72 | 0.01 | 0.18 |
| Rest of FSU | 10.89 | 4.88 | 1.43 | 22.57 | 0.00 | 0.05 |
| Iran | 23.61 | 6.91 | 1.00 | 27.14 | 0.00 | 0.05 |
| UAE | 19.08 | 9.00 | 0.55 | 13.21 | 0.00 | 0.02 |
| Middle East | 101.67 | 38.05 | 4.66 | 53.04 | 0.01 | 0.13 |
| Rest of OPEC | 38.64 | 13.86 | 2.54 | 16.13 | 0.00 | 0.03 |
| Nigeria | 12.42 | 1.03 | 2.46 | 16.46 | 0.00 | 0.01 |
| South Africa | 9.61 | 4.45 | 0.98 | 21.35 | 0.01 | 0.05 |
| Rest of Africa | 35.65 | 15.27 | 8.20 | 67.71 | 0.00 | 0.05 |
| Italy | 139.79 | 52.18 | 2.31 | 40.84 | 0.01 | 0.06 |
| France | 100.14 | 76.93 | 1.59 | 29.30 | 0.00 | 0.03 |
| Germany | 562.77 | 254.54 | 6.65 | 196.84 | 0.03 | 0.34 |
| Spain | 100.54 | 56.20 | 2.23 | 41.41 | 0.01 | 0.08 |
| UK | 139.91 | 79.94 | 2.14 | 42.37 | 0.01 | 0.05 |
| Scandinavia | 86.40 | 49.71 | 1.12 | 27.13 | 0.00 | 0.05 |
| Rest of EU | 121.48 | 68.83 | 2.87 | 61.21 | 0.01 | 0.09 |
| Rest of Europe | 59.14 | 35.66 | 0.97 | 19.57 | 0.00 | 0.03 |
| USA | 715.29 | 504.05 | 9.55 | 431.25 | 0.05 | 0.53 |
| Canada | 35.96 | 22.76 | 1.30 | 32.37 | 0.00 | 0.04 |
| Mexico | 47.79 | 19.39 | 3.68 | 26.43 | 0.00 | 0.04 |
| Rest of Central America | 32.71 | 15.12 | 2.77 | 14.31 | 0.00 | 0.04 |
| Brazil | 60.04 | 30.67 | 4.34 | 44.88 | 0.01 | 0.05 |
| Rest of South America | 81.21 | 33.59 | 4.44 | 42.62 | 0.01 | 0.07 |

## SI 6.2 Data table for Fig.2

|  | Consumption loss (US$b) | | | | | | | | |
| --- | --- | --- | --- | --- | --- | --- | --- | --- | --- |
| Region | Extractive | Food | Fuels | Manufacturing | Construction | Utilities | Transport& tourism | Trade | Services |
| China (Mainland) | 15.47 | 0.00 | 0.00 | 223.03 | 19.04 | 0.75 | 177.62 | 38.67 | 15.56 |
| Rest of East Asia | 18.74 | 4.11 | 8.99 | 136.67 | 15.75 | 4.35 | 66.94 | 23.47 | 38.32 |
| Rest of Asia | 5.98 | 0.00 | 1.66 | 69.40 | 31.91 | 1.09 | 65.06 | 66.70 | 44.76 |
| Middle East and OPEC | 0.45 | 0.00 | 0.54 | 1.11 | 0.29 | 0.26 | 61.90 | 62.97 | 55.48 |
| Russia and FSU | 0.00 | 0.00 | 0.10 | 0.08 | 0.04 | 0.00 | 7.39 | 46.94 | 14.88 |
| Italy and Spain | 1.86 | 2.59 | 0.01 | 35.99 | 12.50 | 0.50 | 46.33 | 69.33 | 71.23 |
| Germany and France | 1.80 | 1.06 | 1.14 | 286.60 | 7.66 | 6.46 | 67.12 | 33.22 | 257.84 |
| Rest of Europe | 3.96 | 0.00 | 0.65 | 19.85 | 5.88 | 0.48 | 125.84 | 88.72 | 161.57 |
| USA | 0.00 | 0.00 | 0.00 | 0.00 | 0.00 | 0.00 | 133.89 | 374.05 | 207.36 |
| Rest of Americas | 0.06 | 0.00 | 0.10 | 2.85 | 0.03 | 0.41 | 58.91 | 71.73 | 123.62 |
| Africa | 0.16 | 0.00 | 0.60 | 0.13 | 0.09 | 0.25 | 15.18 | 22.69 | 18.56 |
| Oceania | 6.59 | 0.00 | 10.36 | 66.08 | 2.81 | 1.37 | 16.46 | 0.00 | 14.14 |
|  | *Income loss (US$b)* | | | | | | | | |
| Region | Extractive | Food | Fuels | Manufacturing | Construction | Utilities | Transport& tourism | Trade | Services |
| China (Mainland) | 5.88 | 0.00 | 0.00 | 102.82 | 10.33 | 0.24 | 98.23 | 21.97 | 9.26 |
| Rest of East Asia | 8.47 | 1.87 | 2.48 | 59.18 | 7.82 | 1.49 | 31.53 | 10.78 | 26.32 |
| Rest of Asia | 2.04 | 0.00 | 0.52 | 25.17 | 14.51 | 0.28 | 22.67 | 22.23 | 37.60 |
| Middle East and OPEC | 0.16 | 0.00 | 0.15 | 0.37 | 0.13 | 0.07 | 20.14 | 19.17 | 27.64 |
| Russia and FSU | 0.00 | 0.00 | 0.03 | 0.03 | 0.02 | 0.00 | 21.10 | 9.26 | 61.78 |
| Italy and Spain | 0.89 | 0.92 | 0.01 | 16.88 | 6.09 | 0.12 | 18.83 | 28.91 | 35.74 |
| Germany and France | 0.73 | 0.41 | 0.34 | 147.05 | 3.83 | 2.80 | 36.17 | 22.89 | 117.26 |
| Rest of Europe | 1.78 | 0.00 | 0.29 | 10.42 | 3.44 | 0.22 | 61.23 | 48.34 | 108.41 |
| USA | 0.00 | 0.00 | 0.00 | 0.00 | 0.00 | 0.00 | 101.49 | 244.89 | 157.68 |
| Rest of Americas | 0.02 | 0.00 | 0.04 | 1.21 | 0.02 | 0.12 | 24.88 | 26.08 | 69.17 |
| Africa | 0.06 | 0.00 | 0.18 | 0.05 | 0.04 | 0.08 | 5.00 | 8.23 | 7.13 |
| Oceania | 2.97 | 0.00 | 4.00 | 33.57 | 1.38 | 0.48 | 9.56 | 0.00 | 9.23 |

|  | Employment loss (million FTE) | | | | | | | | |
| --- | --- | --- | --- | --- | --- | --- | --- | --- | --- |
| Region | Extractive | Food | Fuels | Manufacturing | Construction | Utilities | Transport & tourism | Trade | Services |
| China (Mainland) | 0.68 | 0.00 | 0.00 | 7.63 | 0.55 | 0.02 | 8.03 | 2.71 | 2.63 |
| Rest of East Asia | 0.87 | 0.27 | 0.23 | 2.78 | 0.38 | 0.08 | 1.91 | 0.40 | 1.04 |
| Rest of Asia | 0.41 | 0.00 | 0.13 | 4.82 | 3.65 | 0.04 | 8.11 | 10.41 | 10.07 |
| Middle East and OPEC | 0.01 | 0.00 | 0.01 | 0.03 | 0.05 | 0.00 | 2.30 | 3.21 | 3.14 |
| Russia and FSU | 0.00 | 0.00 | 0.00 | 0.00 | 0.00 | 0.00 | 2.62 | 1.65 | 6.96 |
| Italy and Spain | 0.04 | 0.07 | 0.00 | 0.96 | 0.15 | 0.00 | 0.86 | 1.21 | 1.23 |
| Germany and France | 0.06 | 0.03 | 0.02 | 3.57 | 0.11 | 0.04 | 1.39 | 0.46 | 2.56 |
| Rest of Europe | 0.11 | 0.00 | 0.01 | 0.23 | 0.08 | 0.01 | 2.37 | 1.18 | 3.12 |
| USA | 0.00 | 0.00 | 0.00 | 0.00 | 0.00 | 0.00 | 2.73 | 3.39 | 3.43 |
| Rest of Americas | 0.00 | 0.00 | 0.00 | 0.05 | 0.00 | 0.01 | 3.19 | 4.67 | 8.60 |
| Africa | 0.04 | 0.00 | 0.03 | 0.01 | 0.01 | 0.00 | 1.65 | 5.81 | 4.09 |
| Oceania | 0.12 | 0.00 | 0.13 | 0.86 | 0.06 | 0.01 | 0.31 | 0.00 | 0.25 |
|  | GHG reduction (Mt CO_2_-eq) | | | | | | | | |
| Region | Extractive | Food | Fuels | Manufacturing | Construction | Utilities | Transport & tourism | Trade | Services |
| China (Mainland) | 50.71 | 0.00 | 0.00 | 276.28 | 19.44 | 0.12 | 112.23 | 8.68 | 6.03 |
| Rest of East Asia | 27.37 | 3.50 | 18.26 | 95.85 | 7.38 | 6.70 | 38.71 | 5.02 | 10.01 |
| Rest of Asia | 28.57 | 0.00 | 8.49 | 84.86 | 30.75 | 28.58 | 69.40 | 38.73 | 62.10 |
| Middle East and OPEC | 2.70 | 0.00 | 0.94 | 1.52 | 0.86 | 1.20 | 46.56 | 15.09 | 40.64 |
| Russia and FSU | 0.00 | 0.00 | 6.22 | 0.16 | 0.07 | 0.00 | 26.45 | 21.14 | 50.26 |
| Italy and Spain | 0.80 | 1.17 | 0.06 | 19.91 | 3.04 | 1.94 | 27.11 | 12.62 | 15.59 |
| Germany and France | 2.42 | 0.54 | 1.49 | 122.26 | 2.04 | 24.09 | 35.98 | 4.07 | 33.25 |
| Rest of Europe | 3.98 | 0.00 | 0.71 | 7.65 | 1.88 | 0.78 | 68.58 | 12.57 | 54.14 |
| USA | 0.00 | 0.00 | 0.00 | 0.00 | 0.00 | 0.00 | 344.07 | 30.48 | 56.71 |
| Rest of Americas | 0.25 | 0.00 | 0.07 | 2.22 | 0.02 | 2.32 | 60.94 | 15.69 | 79.10 |
| Africa | 3.52 | 0.00 | 3.83 | 0.33 | 0.18 | 3.05 | 16.99 | 29.03 | 48.58 |
| Oceania | 11.55 | 0.00 | 27.57 | 36.46 | 1.45 | 1.75 | 19.56 | 0.00 | 4.64 |

|  | PM2.5 reduction (kt) | | | | | | | | |
| --- | --- | --- | --- | --- | --- | --- | --- | --- | --- |
| Region | Extractive | Food | Fuels | Manufacturing | Construction | Utilities | Transport & tourism | Trade | Services |
| China (Mainland) | 36.924 | 0.000 | 0.000 | 177.629 | 14.845 | 0.074 | 46.241 | 5.400 | 3.156 |
| Rest of East Asia | 8.476 | 0.996 | 2.500 | 28.885 | 2.971 | 2.871 | 7.169 | 1.838 | 7.026 |
| Rest of Asia | 10.851 | 0.000 | 0.339 | 28.030 | 11.863 | 6.245 | 17.558 | 6.769 | 9.012 |
| Middle East and OPEC | 1.408 | 0.000 | 0.037 | 0.246 | 0.082 | 0.033 | 7.184 | 3.389 | 3.017 |
| Russia and FSU | 0.000 | 0.000 | 0.036 | 0.053 | 0.031 | 0.000 | 3.048 | 3.930 | 5.333 |
| Italy and Spain | 0.210 | 0.141 | 0.009 | 4.321 | 0.547 | 0.212 | 4.922 | 1.889 | 0.917 |
| Germany and France | 0.349 | 0.057 | 0.123 | 23.901 | 0.421 | 0.799 | 5.118 | 0.587 | 3.345 |
| Rest of Europe | 0.843 | 0.000 | 0.094 | 1.753 | 0.395 | 0.269 | 11.627 | 2.239 | 6.554 |
| USA | 0.000 | 0.000 | 0.000 | 0.000 | 0.000 | 0.000 | 44.754 | 3.590 | 3.754 |
| Rest of Americas | 0.036 | 0.000 | 0.010 | 0.341 | 0.004 | 0.092 | 11.693 | 2.086 | 5.823 |
| Africa | 0.154 | 0.000 | 0.251 | 0.134 | 0.060 | 0.812 | 2.703 | 1.523 | 2.690 |
| Oceania | 0.808 | 0.000 | 1.391 | 8.855 | 0.420 | 0.066 | 2.787 | 0.000 | 0.352 |
|  | *SO_2_ & NO_x_ reduction (kt)* | | | | | | | | |
| Region | Extractive | Food | Fuels | Manufacturing | Construction | Utilities | Transport & tourism | Trade | Services |
| China (Mainland) | 55.102 | 0.000 | 0.000 | 970.586 | 68.228 | 0.346 | 282.214 | 26.776 | 9.858 |
| Rest of East Asia | 40.385 | 8.909 | 31.673 | 258.752 | 23.053 | 25.283 | 75.933 | 12.988 | 33.964 |
| Rest of Asia | 18.395 | 0.000 | 6.513 | 263.840 | 92.450 | 216.506 | 138.111 | 73.065 | 50.521 |
| Middle East and OPEC | 0.643 | 0.000 | 0.596 | 2.657 | 2.006 | 2.583 | 110.641 | 55.262 | 57.435 |
| Russia and FSU | 0.000 | 0.000 | 1.098 | 0.795 | 0.356 | 0.000 | 75.312 | 55.894 | 96.198 |
| Italy and Spain | 1.667 | 1.641 | 0.087 | 42.080 | 7.237 | 7.739 | 39.878 | 21.392 | 11.179 |
| Germany and France | 1.615 | 0.768 | 2.425 | 235.193 | 3.787 | 33.087 | 46.592 | 6.905 | 42.246 |
| Rest of Europe | 4.936 | 0.000 | 1.345 | 17.142 | 4.458 | 6.182 | 100.927 | 23.501 | 63.406 |
| USA | 0.000 | 0.000 | 0.000 | 0.000 | 0.000 | 0.000 | 418.489 | 42.403 | 69.151 |
| Rest of Americas | 0.103 | 0.000 | 0.359 | 4.437 | 0.047 | 16.483 | 112.327 | 37.829 | 58.362 |
| Africa | 1.162 | 0.000 | 4.347 | 1.017 | 1.004 | 28.670 | 29.371 | 26.713 | 16.731 |
| Oceania | 9.972 | 0.000 | 33.138 | 120.126 | 5.326 | 8.677 | 36.518 | 0.000 | 4.264 |

## SI 6.3 Production layer decompositions

**PLD by sector**


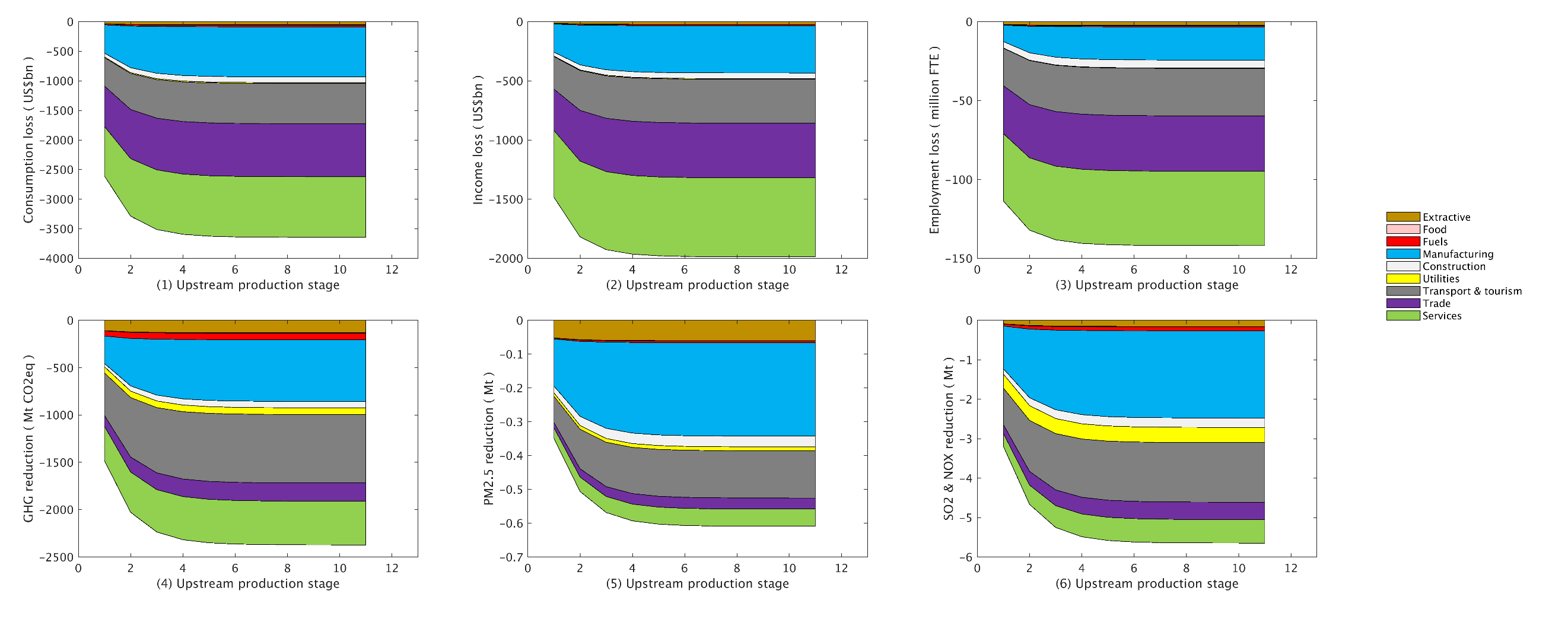


Fig. *SI*9: Production layer decomposition of COVID-19 impacts, by industry sector. The horizontal axis depicts production layers, with ‘1’ being the sectors immediately hit by losses (lockdowns, closures, bans), ‘2’ their suppliers, ‘3’ the suppliers of their suppliers, etc. Transport and tourism are mostly affected.

**PLD by region**


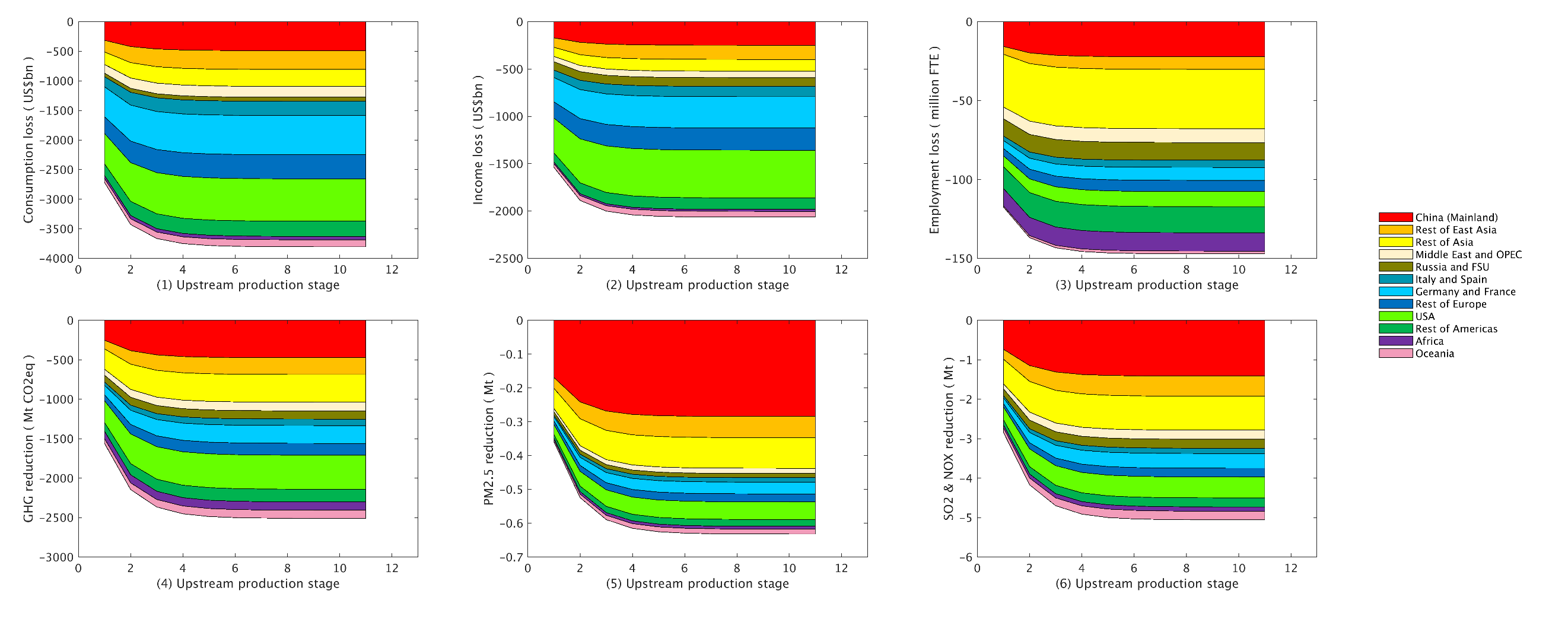


Fig. *SI*10: Production layer decomposition of COVID-19 impacts, by region. The horizontal axis depicts production layers, with ‘1’ being the regions immediately hit by losses (lockdowns, closures, bans), ‘2’ their immediate trade partners, ‘3’ the partners of trade partners, etc. China and East Asia are mostly affected.

## SI 6.4 Average wages of lost employment

Dividing the income embodiment of the consumption loss $\tilde{\mathbf{y}}\boldsymbol{-}\mathbf{y}$ by its employment loss yields average lost wages. The table below shows that these lost wages are not substantially different from average wages in different regions.

| Region | Wage (US$/cap) |
| --- | --- |
| China (Mainland): | 10,975 |
| Rest of East Asia: | 14,177 |
| Rest of Asia: | 3,548 |
| Middle East and OPEC: | 8,433 |
| Russia and FSU: | 8,246 |
| Italy and Spain: | 27,563 |
| Germany and France: | 49,239 |
| Rest of Europe: | 31,202 |
| USA: | 41,041 |
| Rest of Americas: | 7,026 |
| Africa: | 2,3747 |
| Oceania: | 31,387 |

Tab. *SI* 6.3: Average wages of lost employment

## SI 6.5 GHG emissions time series


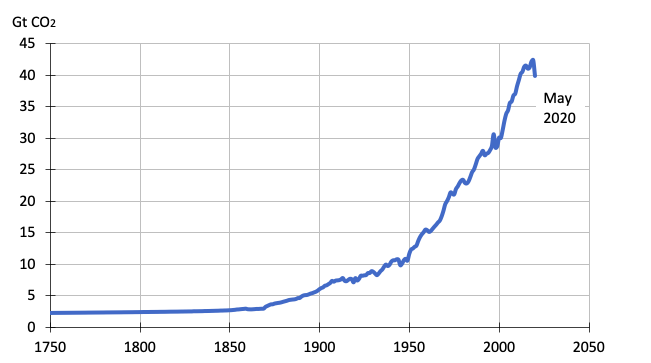


Fig. *SI*11: Time series of total global CO_2_ emissions from 1750 to May 2020. Data from 1750 to 2018 are from the Global Carbon Budget^78^, 2019 is estimated using projection ratios from^19^ and May 2020 data are from this study (which include non-CO_2_ emissions).


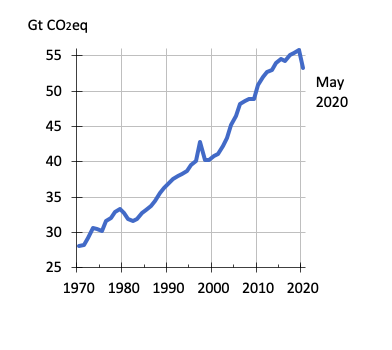


Fig. *SI*12: Time series of total global GHG emissions from 1970 to May 2020 (including CO_2_, CH_4_, N_2_O, F-gases and emissions from land use change and forestry). Data from 1970 to 2017 are from the EDGAR database^19^, 2018 and 2019 are estimated using projection ratios from Peters et al.^79^ and May 2020 data are from this study.


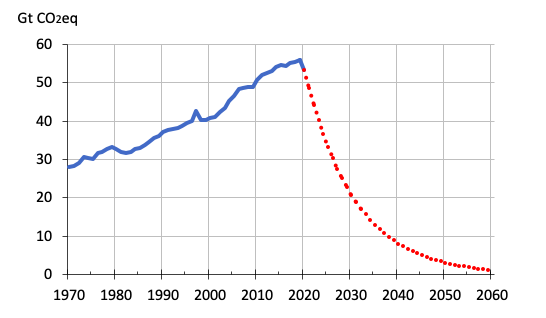

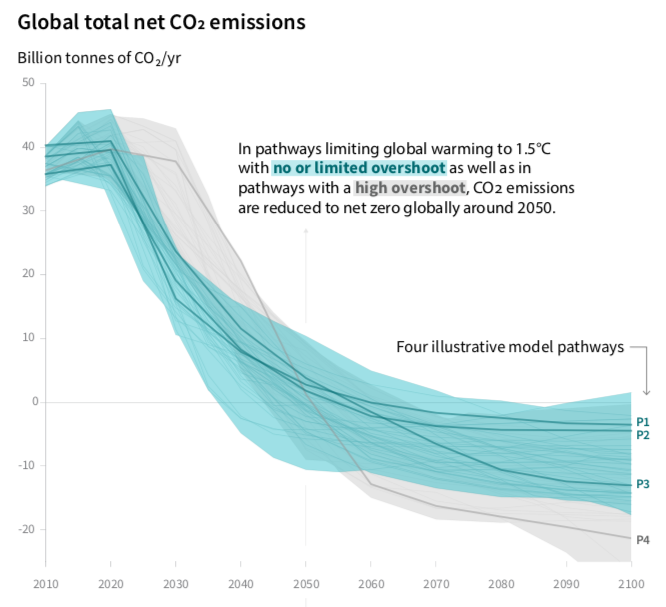


Fig. *SI*13: Left panel: Same as Fig. *SI*12 but with a continuous drop of 9% per year after 2020 visualised in the red, dotted line (i.e. twice the drop of 4.5% induced by COVID-19 until May 2020 estimated in this study). In order to limit global warming to 1.5ºC without the use of technology that removes carbon dioxide from the atmosphere, a drop of about 9-10% in total global GHG emissions would be required *every year* between 2020 and 2050. Right panel: Figure taken from the IPCC 2018 Special Report on Global Warming of 1.5ºC (Summary for Policy Makers^80^, p.15), showing global net anthropogenic CO_2_ emissions in modelled pathways limiting global warming to 1.5°.

## SI 6.6 Supply chain losses as a result of COVID-19


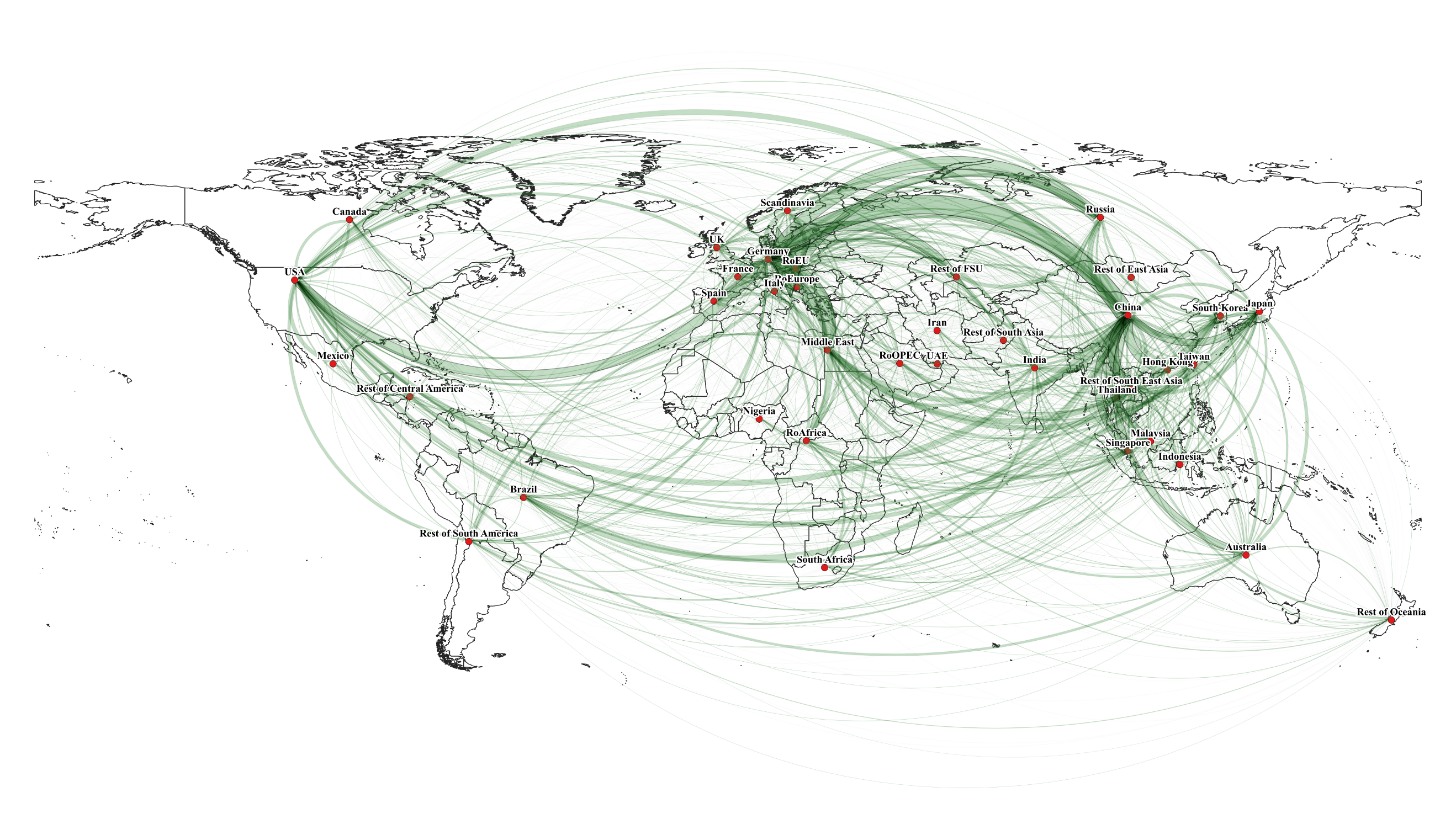


Fig. *SI*14: Reductions in emissions as a result of a decline in trade volume in international supply chains due to the global COVID-19 effects. Lines connect ultimate origins and destinations of supply chains, both direct and multi-node. Line thickness represents trade volume lost.

# **SI 7 – Comparisons with other studies**

Given the fluid nature of the global COVID-19 pandemic, estimates of global consumption, GDP or export losses arrived at from our analysis are necessarily fraught with uncertainty over the future trajectory of the pandemic. Nevertheless, a number of estimates are within similar ranges (*SI* 7.1). Our estimate being within the range of published scenario assessments can serve as an indication for our estimates of environmental gains (GHG, PM_2.5_, SO_2_ and NO_x_) also being of a realistic order of magnitude. This is because, given current technology and its emissions intensities, economic activity and emissions can be expected to be roughly proportional.

## SI 7.1 Consumption and GDP

A number of scenario studies estimate consumption / GDP losses.

| GDP loss (US$bn) | ADB 7/3^81^ | PwC March^82^ | McKinsey A1 24/3^83^ | Bloomberg Scen 4 7/3^84^ | World Bank pandemic^24^ | World Bank amplified pandemic | CAMA Scen S04^85^ | CAMA Scen S06^85^ | this study |
| --- | --- | --- | --- | --- | --- | --- | --- | --- | --- |
| China | 237 | 111 | 254 | 312 | 480 | 560 | 426 | 1618 | 847 |
| East Asia |  | 116 |  | 162 | 168 | 339 | 171 | 676 | 538 |
| Rest of Asia | 42 | 113 |  | 585 | 148 | 366 | 511 | 1964 | 493 |
| Europe |  | 290 | 1330 | 785 | 403 | 839 | 459 | 1818 | 1117 |
| Americas |  | 381 |  | 700 | 476 | 961 | 563 | 2326 | 1363 |
| Middle East & North Africa |  | 47 |  | 60 | 59 | 126 | 118 | 452 | 203 |
| Rest of Africa |  | 21 |  |  | 29 | 59 | 54 | 214 | 58 |
| Oceania |  | 26 |  | 42 | 29 | 58 | 27 | 103 | 74 |
| Total | **347** | **1105** | **2656** | **2645** | **1791** | **3308** | **2329** | **9171** | **4692** |

Tab. *SI*7.1: Comparisons of the impacts of the COVID-19 pandemic, for loss of GDP. The Bloomberg, World Bank and CAMA scenarios are all of global pandemics.

The International Monetary Fund projects that the global economy will experience the worst recession since the Great Depression, with global growth projected to fall by 3% ^86^.

## SI 7.2 Exports

China’s custom data for January and February report a loss of exports of about 17% on the year prior ^87-89^, which equates to a loss of just above US$ 60 billion. For comparison, our analysis yields a loss of US$ 143 billion for the whole year.

## SI 7.3 Employment

On 29 April 2020, the International Labour Organization (ILO) released modelling results, reporting 1^st^-quarter global job losses of 130 million FTE, and 2^nd^-quarter global job losses of 305 million FTE (Tab. *SI* 7.3). On a full-year basis, these two figures represent global job losses of 110 million FTE. These figures are subject to assumptions about the length of the working week, as is evident from two ILO estimates from 7 April 2020, using 40- and 48-hour weeks, respectively.

Also according to the ILO, about 1.6 billion workers, almost half of the global workforce, were “significantly affected” by lockdown measures ^90^. Assuming that these workers are out of work for 6 weeks yields a global loss of employment of 180 million FTE.

Against the information provided by the ILO, our estimate of 147 million FTE for the February-to-May period appears realistic.

| Employment loss  (million FTE) | ILO  2^nd^ qtr. 2020,  48-hr week  7/4 ^91^ | ILO  same, but  for 40-hr week  7/4 ^91^ | ILO  1^st^ qtr. 2020,  48-hr week  29/4^90^ | ILO  2^nd^ qtr. 2020,  48-hr week  29/4^90^ | this study,  data for  Feb-May |
| --- | --- | --- | --- | --- | --- |
| China |  |  |  |  | 22 |
| East Asia | 125 | 150 |  |  | 8 |
| Rest of Asia |  |  |  |  | 38 |
| Europe | 20 | 24 |  |  | 31 |
| Americas | 24 | 29 |  |  | 26 |
| Middle East & North Africa | 5 | 6 |  |  | 9 |
| Rest of Africa | 19 | 22 |  |  | 12 |
| Oceania | incl in Asia | incl in Asia |  |  | 1.7 |
| Quarterly estimates | **195** | **230** | **130** | **305** |  |
| Whole-of-year equivalent | **49** | **58** | **110** | | **147** |

Tab. *SI* 7.3: Comparisons of the impacts of the COVID-19 pandemic, for employment.

# **References**

1 worldometer. *COVID-19 Coronavirus Pandemic*, <https://[www.worldometers.info/coronavirus/](http://www.worldometers.info/coronavirus/)> (2020).

2 Lenzen, M. *et al.* The Global MRIO Lab - charting the world economy. *Economic Systems Research* **29**, 158-186, doi:10.1080/09535314.2017.1301887 (2017).

3 Lenzen, M. *et al.* Compiling and using input-output frameworks through collaborative virtual laboratories. *Science of the Total Environment* **485–486**, 241–251 (2014).

4 Lenzen, M., Kanemoto, K., Moran, D. & Geschke, A. Mapping the Structure of the World Economy. *Environmental Science & Technology* **46**, 8374-8381, doi:10.1021/es300171x (2012).

5 Lenzen, M., Moran, D., Kanemoto, K. & Geschke, A. Building Eora: A global multi-region input-output database at high country and sector resolution. *Economic Systems Research* **25**, 20-49 (2013).

6 Lenzen, M., Gallego, B. & Wood, R. Matrix balancing under conflicting information. *Economic Systems Research* **21**, 23-44 (2009).

7 Geschke, A., Ugon, J., Lenzen, M., Kanemoto, K. & Moran, D. D. Balancing and reconciling large multi-regional input–output databases using parallel optimisation and high-performance computing. *Journal of Economic Structures* **8**, 2, doi:10.1186/s40008-019-0133-7 (2019).

8 UNSD. National Accounts Main Aggregates Database. Report No. https://unstats.un.org/unsd/snaama/, (United Nations Statistics Division, New York, USA, 2020).

9 UNSD. Available Classifications. Report No. <http://unstats.un.org/unsd/cr/registry/regct.asp?Lg=1>, (United Nations Statistics Division, New York, USA, 2007).

10 UNSD. National Accounts Official Data. Report No. <http://data.un.org/Browse.aspx?d=SNA>, (United Nations Statistics Division, New York, USA, 2019).

11 UNIDO. Industrial Statistics Database at the 4-digit level of ISIC (INDSTAT4). Report No. <http://www.unido.org/resources/statistics/statistical-databases.html>, (United Nations, New York, USA, 2019).

12 UNSD. UN comtrade - United Nations Commodity Trade Statistics Database. Report No. <http://comtrade.un.org/>, (United Nations Statistics Division, UNSD, New York, USA, 2019).

13 UNSD. UN ServiceTrade. Report No. https://unstats.un.org/unsd/servicetrade/default.aspx, (United Nations Statistics Division, UNSD, New York, USA, 2019).

14 Lenzen, M., Kanemoto, K., Moran, D. & Geschke, A. Mapping the structure of the world economy. *Environmental Science & Technology* **46**, 8374–8381, <http://dx.doi.org/8310.1021/es300171x> (2012).

15 World Bank. GDP growth (annual %). Report No. https://data.worldbank.org/indicator/NY.GDP.MKTP.KD.ZG, (World Bank, Washington D.C., USA, 2020).

16 ILO. *LABORSTA—Main statistics (annual): Employment general level, by economic activity, by occupation, by status in employment. Geneva: International Labor Organization.*, <<http://laborsta.ilo.org>> (2015).

17 Alsamawi, A., Murray, J. & Lenzen, M. The employment footprints of nations: Uncovering master-servant relationships. *Journal of Industrial Ecology* **18**, 59-70, doi:10.1111/jiec.12104 (2014).

18 Global Warming Potential Values (AR5). *Greenhouse Gas Protocol*, <https://[www.ghgprotocol.org/sites/default/files/ghgp/Global-Warming-Potential-Values%20%28Feb%2016%202016%29_1.pdf](http://www.ghgprotocol.org/sites/default/files/ghgp/Global-Warming-Potential-Values%20%28Feb%2016%202016%29_1.pdf)> (2016).

19 Janssens-Maenhout, G. *et al.* EDGAR v4.3.2 Global Atlas of the three major Greenhouse Gas Emissions for the period 1970-2012. *Earth Syst. Sci. Data Discuss.* **2017**, 1-55, doi:10.5194/essd-2017-79 (2017).

20 Acero, A. P., Rodríguez, C. & Ciroth, A. Impact assessment methods in Life Cycle Assessment and their impact categories. Report No. https://[www.openlca.org/wp-content/uploads/2015/11/openLCA_LCIA_METHODS-v.1.5.6.pdf](http://www.openlca.org/wp-content/uploads/2015/11/openLCA_LCIA_METHODS-v.1.5.6.pdf), (GreenDelta, 2017).

21 GHK. in *A study to examine the benefits of the End of Life Vehicles Directive and the costs and benefits of a revision of the 2015 targets for recycling, re-use and recovery under the ELV Directive* (ed DG Environment) (European Commission, 2006).

22 World Bank. *GDP growth (annual %)*, <https://data.worldbank.org/indicator/NY.GDP.MKTP.KD.ZG> (2020).

23 Ensheng, D., Hongru, D. & Lauren, G. An interactive web-based dashboard to track COVID-19 in real time. *The Lancet Infectious Diseases*, doi:https://doi.org/10.1016/S1473-3099(20)30120-1 (2020).

24 World Bank. World Bank East Asia and Pacific Economic Update, April 2020 : East Asia and Pacific in the Time of COVID-19. (World Bank, Washington, DC, 2020).

25 Aguiar, A., Chepeliev, M., Corong, E. L., McDougall, R. & van der Mensbrugghe, D. The GTAP data base: Version 10. *Journal of Global Economic Analysis* **4**, 1-27 (2019).

26 OAG. *Global Scheduled Flights Change year-over-year Chicago*, <https://[www.oag.com/coronavirus-airline-schedules-data](http://www.oag.com/coronavirus-airline-schedules-data)> (2020).

27 UNSD-EUROSTAT-OECD-WTO. *Tourism Satellite Account: Recommended Methodological Framework*. (World Tourism Organization, 2008).

28 IATA. *IATA Fact Sheet Montreal*, <https://[www.iata.org/contentassets/f05be6a9ea4f439aa6b99a6717ccfbb0/fact-sheet-iata-1.pdf](http://www.iata.org/contentassets/f05be6a9ea4f439aa6b99a6717ccfbb0/fact-sheet-iata-1.pdf)> (2020).

29 IATA. *COVID 19 Updated Impact Assessment*, <https://[www.iata.org/en/iata-repository/publications/economic-reports/third-impact-assessment/](http://www.iata.org/en/iata-repository/publications/economic-reports/third-impact-assessment/)> (2020).

30 Aljazerra. *Coronavirus: Travel restrictions, border shutdowns by country 2020*, <https://[www.aljazeera.com/news/2020/05/saudi-activists-khashoggi-murder-case-political-personal-200522103715386.html](http://www.aljazeera.com/news/2020/05/saudi-activists-khashoggi-murder-case-political-personal-200522103715386.html)> (2020).

31 GardWorld. *News Alerts 2020*, <https://[www.garda.com/crisis24/news-alerts](http://www.garda.com/crisis24/news-alerts)> (2020).

32 IATA. *Government Measures Related to Coronavirus (COVID-19)*, <https://[www.iata.org/en/programs/safety/health/diseases/government-measures-related-to-coronavirus/?page=1&search=&ordering=DateDesc](http://www.iata.org/en/programs/safety/health/diseases/government-measures-related-to-coronavirus/?page=1&search=&ordering=DateDesc)> (2020).

33 Think Global Health. *Travel Restrictions on China due to COVID-19*, <https://[www.thinkglobalhealth.org/article/travel-restrictions-china-due-covid-19](http://www.thinkglobalhealth.org/article/travel-restrictions-china-due-covid-19)> (2020).

34 UNECE. *Observatory on Border Crossings Status due to COVID-19 Home*, <https://wiki.unece.org/display/CTRBSBC/Observatory+on+Border+Crossings+Status+due+to+COVID-19+Home> (2020).

35 WorldAware. *COVID-19 Risk Intelligence and Resource Cente*, <https://[www.worldaware.com/covid-19-risk-intelligence-and-resource-center](http://www.worldaware.com/covid-19-risk-intelligence-and-resource-center)> (2020).

36 TravelDaily. *季度国内旅游人次降五成，三地推行2.5天周末假刺激消费*, < https://[www.traveldaily.cn/article/136686](http://www.traveldaily.cn/article/136686)> (2020).

37 Bullard, C. W. & Sebald, A. V. Monte Carlo sensitivity analysis of input-output models. *The Review of Economics and Statistics* **LXX**, 708-712 (1988).

38 Hale, T., Webster, S., Petherick, A., Phillips, T. & Kira, B. *Oxford COVID-19 Government Response Tracker. Data use policy: Creative Commons Attribution CC BY standard*, 2020).

39 Lau, J. *Governments have ‘responsibility’ to help reimburse students*, <https://[www.timeshighereducation.com/news/governments-have-responsibility-tohelp-reimburse-students](http://www.timeshighereducation.com/news/governments-have-responsibility-tohelp-reimburse-students)> (2020).

40 Cook, L. & Warwick-Ching, L. *Financial Times*, <https://[www.ft.com/content/21928dda-e2f0-49f3-8f97-c82831983025](http://www.ft.com/content/21928dda-e2f0-49f3-8f97-c82831983025)> (2020).

41 MIUR. *Italian Government - Ministero dell'Istruzione,  dell'Università e della Ricerca:  Coronavirus, da salvaguardia anno scolastico a rimborsi viaggi istruzione: le ultime misure in materia di scuola e università*, <https://[www.miur.gov.it/web/guest/-/coronavirus-da-salvaguardia-anno-scolastico-a-rimborsi-viaggi-istruzione-le-ultime-misure-in-materia-di-scuola-e-universita](http://www.miur.gov.it/web/guest/-/coronavirus-da-salvaguardia-anno-scolastico-a-rimborsi-viaggi-istruzione-le-ultime-misure-in-materia-di-scuola-e-universita)> (2020).

42 IHE. *Latest Coronavirus News  - How higher education is reacting to the new coronavirus pandemic.*, <https://[www.insidehighered.com/news/2020/04/01/live-updates-latest-news-coronavirus-and-higher-education](http://www.insidehighered.com/news/2020/04/01/live-updates-latest-news-coronavirus-and-higher-education)> (2020).

43 Newcastle University. *Coronavirus Frequently Asked Questions*, <https://[www.ncl.ac.uk/wellbeing/coronavirus-updatedinformation/faq/#general](http://www.ncl.ac.uk/wellbeing/coronavirus-updatedinformation/faq/#general)> (2020).

44 Stacey, V. *Language schools fear losses due to COVID-19 outbreak across Italy*, <https://thepienews.com/news/schools-fear-losses-due-to-covid-19-outbreak-in-italy/> (2020).

45 O'Brien, J. *My child is staying home from school because of coronavirus. Is that illegal?*, <https://theconversation.com/my-child-is-staying-home-from-school-because-of-coronavirus-is-that-illegal-134245> (2020).

46 Burki, T. K. *COVID-19: consequences for higher education*, <https://doi.org/10.1016/S1470-2045(20)30287-4> (2020).

47 World Economic Forum. *How COVID-19 is driving a long-overdue revolution in education*, <https://[www.weforum.org/agenda/2020/05/how-covid-19-is-sparking-a-revolution-in-higher-education/](http://www.weforum.org/agenda/2020/05/how-covid-19-is-sparking-a-revolution-in-higher-education/)> (2020).

48 Steenge, A. E. & Bočkarjova, M. Thinking about imbalances in post-catastrophe economies: An input–output based proposition. *Economic Systems Research* **19**, 205-223, doi:10.1080/09535310701330308 (2007).

49 Dietzenbacher, E., van Burken, B. & Kondo, Y. Hypothetical extractions from a global perspective. *Economic Systems Research* **31**, 505-519, doi:10.1080/09535314.2018.1564135 (2019).

50 Schulte in den Bäumen, H., Moran, D., Lenzen, M., Cairns, I. & Steenge, A. How severe space weather can disrupt global supply chains. *Natural Hazards and Earth System Science* **14**, 2749-2759, doi:10.5194/nhess-14-2749-2014 (2014).

51 Schulte in den Bäumen, H., Többen, J. & Lenzen, M. Labour forced impacts and production losses due to the 2013 flood in Germany. *Journal of Hydrology* **527**, 142-150 (2015).

52 Lenzen, M. *et al.* Economic damage and spill-overs from a tropical cyclone. *Nat. Hazards Earth Syst. Sci. Discuss.* **2018**, 1-28, doi:10.5194/nhess-2017-440 (2018).

53 Faturay, F. *et al.* Using Virtual Laboratories for disaster analysis – A case study of Taiwan. *Economic Systems Research* **32**, 58-83 (2020).

54 Malik, A. *et al.* Impacts of climate change on food supply – a case study of New South Wales, Australia. submitted (2020).

55 Lenzen, M. *et al.* The carbon footprint of global tourism. *Nature Climate Change* **8**, 522 (2018).

56 Heijungs, R. & Lenzen, M. Error propagation methods for LCA—a comparison. *The International Journal of Life Cycle Assessment* **19**, 1445-1461, doi:10.1007/s11367-014-0751-0 (2014).

57 Imbeault-Tétreault, H., Jolliet, O., Deschênes, L. & Rosenbaum, R. K. Analytical Propagation of Uncertainty in Life Cycle Assessment Using Matrix Formulation. *J Ind Ecol* **17**, 485-492, doi:10.1111/jiec.12001 (2013).

58 Lloyd, S. M. & Ries, R. Characterizing, Propagating, and Analyzing Uncertainty in Life-Cycle Assessment: A Survey of Quantitative Approaches. *J Ind Ecol* **11**, 161-179, doi:10.1162/jiec.2007.1136 (2007).

59 Lenzen, M. Aggregation versus disaggregation in input-output analysis of the environment. *Economic Systems Research* **23**, 73 – 89 (2011).

60 Bullard, C. W. & Sebald, A. V. Effects of parametric uncertainty and technological change on input-output models. *Review of Economics and Statistics* **LIX**, 75-81 (1977).

61 Hondo, H., Sakai, S. & Tanno, S. Sensitivity analysis of total CO_2_ emission intensities estimated using an input-output table. *Appl Energ* **72**, 689-704 (2002).

62 Malik, A., Lenzen, M., McAlister, S. & McGain, F. The carbon footprint of Australian health care. *The Lancet Planetary Health* **2**, e27-e35, doi:10.1016/S2542-5196(17)30180-8 (2018).

63 Lenzen, M. *et al.* The carbon footprint of global tourism. *Nature Climate Change*, doi:10.1038/s41558-018-0141-x (2018).

64 Lenzen, M., Wood, R. & Wiedmann, T. Uncertainty analysis for Multi-Region Input-Output models – a case study of the UK’s carbon footprint. *Economic Systems Research* **22**, 43-63 (2010).

65 De Salazar, P. M., Niehus, R., Taylor, A., Buckee, C. O. & Lipsitch, M. Using predicted imports of 2019-nCoV cases to determine locations that may not be identifying all imported cases. *medRxiv*, 2020.2002.2004.20020495, doi:10.1101/2020.02.04.20020495 (2020).

66 Jones, S. & Mohdin, A. Coronavirus: Iran denies cover-up as six deaths reported in Italy. *The Guardian* (2020).

67 RSF. Turkmenistan bans the word “coronavirus”. Report No. https://rsf.org/en/news/turkmenistan-bans-word-coronavirus, (Reporters Without Borders, Paris, France, 2020).

68 AFP. North Korea's coronavirus-free claim met with scepticism. *The Guardian* (2020).

69 Lenzen, M., Kanemoto, K., Moran, D. & Geschke, A. Uncertainty and Reliability in the Eora MRIO tables. Report No. <http://globalcarbonfootprint.com/EoraConfidence.pdf>, (Sydney, Australia, 2012).

70 Tukker, A. & Dietzenbacher, E. Global multiregional input-output frameworks: An introduction and outlook. *Economic Systems Research* **25**, 1-19, doi:10.1080/09535314.2012.761179 (2013).

71 Moran, D. & Wood, R. Convergence between the Eora, WIOD, EXIOBASE, and OpenEU's consumption-based carbon accounts. *Economic Systems Research* **26**, 245-261, doi:10.1080/09535314.2014.935298 (2014).

72 Inomata, S. & Owen, A. Comparative evaluation of MRIO databases. *Economic Systems Research* **26**, 239-244, doi:10.1080/09535314.2014.940856 (2014).

73 Steen-Olsen, K., Owen, A., Hertwich, E. G. & Lenzen, M. Effects of sectoral aggregation on CO2 multipliers in MRIO analyses. *Economic Systems Research* **26**, 284-302 (2014).

74 Wiebe, K. S. & Lenzen, M. To RAS or not to RAS? What is the difference in outcomes in multi-regional input–output models? *Economic Systems Research* **28**, 383-402, doi:10.1080/09535314.2016.1192528 (2016).

75 Owen, A., Steen-Olsen, K., Barrett, J., Wiedmann, T. & Lenzen, M. A structural decomposition approach to comparing MRIO databases. *Economic Systems Research* **26**, 262-283 (2014).

76 Owen, A., Wood, R., Barrett, J. & Evans, A. Explaining value chain differences in MRIO databases through structural path decomposition. *Economic Systems Research* **28**, 1-30, doi:10.1080/09535314.2015.1135309 (2016).

77 IATA. Climate Change. Report No. https://[www.iata.org/en/policy/environment/climate-change/](http://www.iata.org/en/policy/environment/climate-change/), (2020).

78 Friedlingstein, P. *et al.* Global carbon budget 2019. *Earth System Science Data* **11**, 1783-1838 (2019).

79 Peters, G. *et al.* Carbon dioxide emissions continue to grow amidst slowly emerging climate policies. *Nature Climate Change* **10**, 3-6 (2020).

80 IPCC. *Special Report: Global Warming of 1.5 ºC. Summary for Policy Makers*, <https://[www.ipcc.ch/sr15/](http://www.ipcc.ch/sr15/)> (2018).

81 ADB. The Economic Impact of the COVID-19 Outbreak on Developing Asia. (Asian Development Bank, 2020).

82 Thorpe, J., Loughridge, J. & Picton, M. The possible economic consequences of a novel coronavirus (COVID-19) pandemic. Report No. March 2020, (PricewaterhouseCoopers, 2020).

83 McKinsey. COVID-19: Briefing materials. (McKinsey & Co., 2020).

84 Orlik, T., Rush, J., Cousin, M. & Hong, J. Coronavirus Could Cost the Global Economy $2.7 Trillion. Here’s How., (Bloomberg, 2020).

85 McKibbin, W. J. & Fernando, R. The global macroeconomic impacts of covid-19: Seven scenarios. Report No. 19/2020, (CAMA, Australian National University, Canberra, Australia, 2020).

86 IMF. *Coronavirus: 'World faces worst recession since Great Depression'*, <https://[www.bbc.com/news/business-52273988](http://www.bbc.com/news/business-52273988)> (2020).

87 Bajpai, P. *An Overview of China's Economy and How Coronavirus Affected It*, 2020).

88 Bermingham, F. Coronavirus: China’s exports and imports plummet in January and February. *South China Morning Post*, 7 March (2020).

89 General Administration of Customs & Summary of Imports and Exports (In USD) A: Annually, January-February. Report No. <http://english.customs.gov.cn/statics/report/monthly.html>, (General Administration of Customs of the People’s Republic of China, Beijing, China, 2020).

90 ILO. *ILO Monitor: COVID-19 and the world of work. Third edition*, <https://[www.ilo.org/wcmsp5/groups/public/---dgreports/---dcomm/documents/briefingnote/wcms_743146.pdf](http://www.ilo.org/wcmsp5/groups/public/---dgreports/---dcomm/documents/briefingnote/wcms_743146.pdf)> (2020).

91 ILO. COVID-19 and the world of work. Report No. https://[www.ilo.org/wcmsp5/groups/public/---dgreports/---dcomm/documents/briefingnote/wcms_740877.pdf](http://www.ilo.org/wcmsp5/groups/public/---dgreports/---dcomm/documents/briefingnote/wcms_740877.pdf), (International Labour Organization, 2020).
